# Exogenous L-fucose attenuates depression induced by chronic unpredictable stress: Implicating core fucosylation has an antidepressant potential

**DOI:** 10.1016/j.jbc.2025.108230

**Published:** 2025-01-27

**Authors:** Dan Wang, Tomohiko Fukuda, Tiangui Wu, Xing Xu, Tomoya Isaji, Jianguo Gu

**Affiliations:** 1 Division of Regulatory Glycobiology, Graduate School of Pharmaceutical Sciences, Tohoku Medical and Pharmaceutical University, Sendai, Miyagi, Japan; 2 Institute of Molecular Biomembrane and Glycobiology, Tohoku Medical and Pharmaceutical University, Sendai, Miyagi, Japan

**Keywords:** core fucosylation, depression, L-fucose, Fut8, neuroinflammation

## Abstract

Core fucosylation is one of the most essential modifications of the *N*-glycans, catalyzed by α1,6-fucosyltransferase (Fut8), which transfers fucose from guanosine 5′-diphosphate (GDP)-fucose to the innermost *N*-acetylglucosamine residue of *N*-glycans in an α1-6 linkage. Our previous studies demonstrated that lipopolysaccharide (LPS) can induce a more robust neuroinflammatory response in *Fut8* homozygous knockout (KO) (*Fut8*^−/−^) and heterozygous KO (*Fut8*^+/−^) mice contrasted to the wild-type (*Fut8*^+/+^) mice. Exogenous administration of L-fucose suppressed LPS-induced neuroinflammation. Numerous studies indicate that neuroinflammation plays a vital role in the development of depression. Here, we investigated whether core fucosylation regulates depression induced by chronic unpredictable stress (CUS), a well-established model for depression. Our results showed that *Fut8*^+/−^ mice exhibited depressive-like behaviors and increased neuroinflammation earlier than *Fut8*^+/+^ mice. Administration of L-fucose significantly reduced CUS-induced depressive-like behaviors and pro-inflammatory cytokine levels in *Fut8*^+/−^ mice. The L-fucose treatment produced antidepressant effects by attenuating the complex formation between gp130 and the interleukin-6 (IL-6) receptor and the JAK2/STAT3 signaling pathway. Notably, L-fucose treatment increased dendritic spine density and postsynaptic density protein 95 (PSD-95) expression, which were suppressed in CUS-induced depression. Furthermore, the effects of L-fucose on the CUS-induced depression were also observed in *Fut8*^+/+^ mice. Our results clearly demonstrate that L-fucose ameliorates neuroinflammation and synaptic defects in CUS-induced depression, implicating that core fucosylation has significant anti-neuroinflammatory activity and an antidepressant potential.

Major depressive disorder (MDD) is a common mental disorder characterized by persistent symptoms of depressed mood and anhedonia (1, 2). MDD heavily impacts patients' sleep quality, appetite, cognitive flexibility, and mobility (3, 4, 5). In severe cases, individuals with depression may exhibit suicidal tendencies (6). Approximately 300 million people worldwide suffer from MDD, and its prevalence continues to rise (7). The World Health Organization listed MDD as the third largest disease burden globally in 2018, with projections indicating it will rank first by 2030 (8).

Numerous studies have shown that MDD is closely related to elevated levels of neuroinflammatory biomarkers, such as interleukin (IL)-6 (9, 10, 11), IL-1β (12, 13), and tumor necrosis factor-alpha (TNF-α) (14, 15, 16). Inflammation has even been proposed as a biomarker in the treatment of depression (17). Rodent models of chronic stress also exhibit increased levels of pro-inflammatory cytokines (18, 19, 20). Depression is increasingly recognized as a microglial disease (21). As immune effector cells in the central nervous system (CNS), microglia play a crucial role in neuroprotection. In depression and its animal models, microglia become overactivated, accelerating the neuroinflammatory response by secreting large amounts of pro-inflammatory (22, 23, 24) cytokines. Excessive inflammation can lead to neuronal apoptosis and cell death (25, 26).

L-fucose is a dietary deoxyhexose sugar that is taken up and processed in cells, converted into guanosine 5′-diphosphate (GDP)-fucose, which is utilized in the endoplasmic reticulum or Golgi by 13 fucosyltransferases to synthesize fucosylated glycans (27, 28, 29). GDP-fucose can be synthesized in two ways: the *de novo* pathway and the salvage pathway, and the *de novo* pathway is dominant (30, 31). Dietary intake or lysosomal breakdown of glycoproteins can provide L-fucose in the salvage pathway. When the *de novo* pathway is compromised, the salvage pathway supplements GDP-fucose to maintain normal physiological functions (32). L-fucose has shown anti-tumor and anti-inflammatory effects in various diseases. For example, in melanoma models, dietary L-fucose enhanced immunotherapy response; it has also been shown to reduce gut inflammation and mitigate high-salt diet-induced inflammation (33, 34, 35). Ample evidence suggests that L-fucose, the predominant bioactive component of fucoidan, possesses neuroprotective and neurogenic effects in the CNS (36, 37, 38). However, the underlying molecular mechanisms for these effects remain unclear.

Core fucosylation is a process catalyzed by α1,6-fucosyltransferase (Fut8), which transfers a fucose residue from GDP-fucose to the innermost *N*-acetylglucosamine residue of *N*-linked glycans in complex and hybrid glycoproteins (39, 40). Patients with FUT8-congenital disorders of glycosylation, characterized by impaired core fucosylation, often present with epilepsy, short stature, microcephaly, and intellectual disabilities (41). Our previous studies demonstrated that *Fut8* homozygous knockout (KO) (*Fut8*^−/−^) mice exhibit memory impairments and abnormal behaviors consistent with schizophrenia-like phenotypes (42), along with decreased hippocampal long-term potentiation (LTP) compared to the wild-type (*Fut8*^+/+^) mice (43). Additionally, Fut8 deficiency *via* shRNA has been shown to impair memory, learning, and neurogenesis in adult mice (44). These findings suggest that a lack of core fucosylation may contribute to developing CNS disorders. Both *Fut8*^−/−^ mice and *Fut8*^+/−^ mice display heightened microglial activation and a more severe inflammatory response to lipopolysaccharide (LPS) stimulation than *Fut8*^+/+^ mice (45, 46), indicating that neuroinflammation may be negatively regulated by core fucosylation. Lack of the *Fut8* gene significantly enhances the complex formation between the IL-6 receptor (IL-6R) and glycoprotein 130 (gp130), resulting in stronger neuroinflammation. Conversely, L-fucose administration increases core fucosylation of gp130, thereby reducing the complex formation and exerting anti-neuroinflammation effects (45). Given the close relationship between neuroinflammation and CNS disorders, we hypothesize that core fucosylation may also influence the onset and progression of depression. In this study, we investigated the effects of core fucosylation on depression induced by chronic unpredictable stress (CUS), one of the most established models for depression (47). Our findings indicate that *Fut8*^+/−^ mice develop depressive-like behaviors earlier than *Fut8*^+/+^ mice, which can be rescued by L-fucose administration. Furthermore, L-fucose ameliorated neuroinflammation and synaptic defects in CUS-induced depression mice. Therefore, L-fucose may hold potential as a therapeutic agent for CNS diseases.

## Results

### *Fut8*^+/−^ mice exhibited depression-like behavior earlier than *Fut8*^+/+^ mice

**Figure 1:**
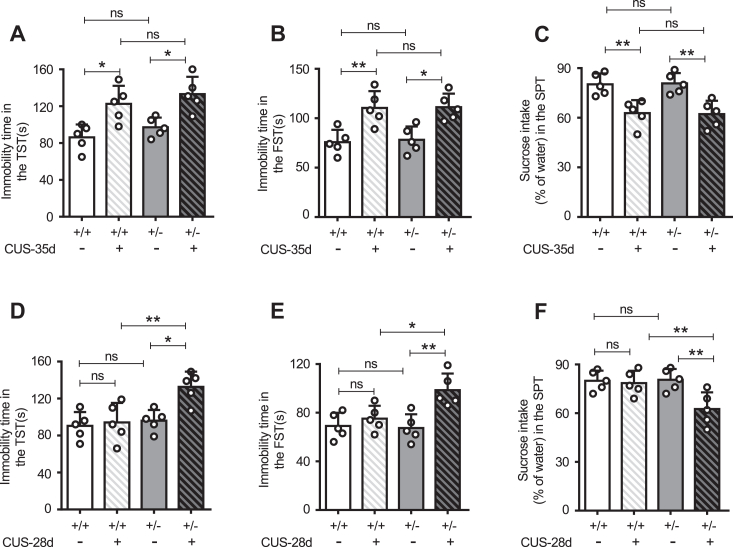
**Comparison of depression-like behaviors upon CUS treatment between *Fut8***^**+/−**^**and *Fut8***^**+/+**^**mice, Chronic unpredictable stress (CUS) models were established in *Fut8***^**+/+**^**and *Fut8***^**+/−**^**mice, grouped at 7, 14, 21, 28, and 35 days post-CUS.** Behavioral assessments were conducted using the tail suspension test (TST) (*A*), forced swimming test (FST) (*B*), and sucrose preference test (SPT) (*C*) after 35 days of CUS. Data for TST (*D*), FST (*E*), and SPT (*F*) at 28 days of CUS are also presented. Data were analyzed by one-way ANOVA with Tukey's multiple comparison tests and shown as mean ± SD. n = 5 mice per group. n.s. *p* > 0.05; ∗*p* < 0.05; ∗∗*p* < 0.01.

Our previous studies revealed that core fucosylation negatively regulates the neuroinflammation induced by LPS; specifically, lower core fucosylation can induce severe neuroinflammation (45, 46). Given the established link between neuroinflammation and depression (12, 15), we explored whether core fucosylation affects the onset and progression of depression. Stress is a significant risk factor for several psychiatric illnesses, including MDD, and is often a trigger for depressive episodes (48, 49, 50). The CUS model of depression is a valid, reliable, and widely used model (47), and the method for simulating the stress variations is quite similar to what humans are exposed to in their daily lives (51, 52). Following 7, 14, 21, 28, and 35 days of exposure to the CUS model, we tested the depressive behavior of *Fut8*^+/+^ and *Fut8*^+/−^ mice. At 35 days, compared to each control mice without CUS, both *Fut8*^+/+^ and *Fut8*^+/−^ CUS mice exhibited increased immobility times in the tail suspension test (TST) (Fig. 1*A*) and forced swimming test (FST) (Fig. 1*B*), as well as decreased sucrose intake in the sucrose preference test (SPT) (Fig. 1*C*), indicating the success of the CUS model. Notably, in the CUS-28-days group, *Fut8*^+/−^ mice showed significantly higher immobility time in the TST (Fig. 1*D*) and FST (Fig. 1*E*) than controls without CUS. In addition, *Fut8*^+/−^ mice exhibited significant attenuation in sucrose preference at 28 days of CUS compared to controls in the SPT (Fig. 1*F*). However, these differences were not observed in *Fut8*^+/+^ mice at 28 days. All mice did not display depressive-like behaviors after 7, 14, and 21 days of CUS (Fig. S1, *A*–*I*). These findings demonstrate that the mice with lower core fucosylation are more susceptible to exhibiting depressive-like behaviors.

### L-fucose administration abolished increased depressive-like behaviors induced by CUS in *Fut8*^+/−^ mice

**Figure 2:**
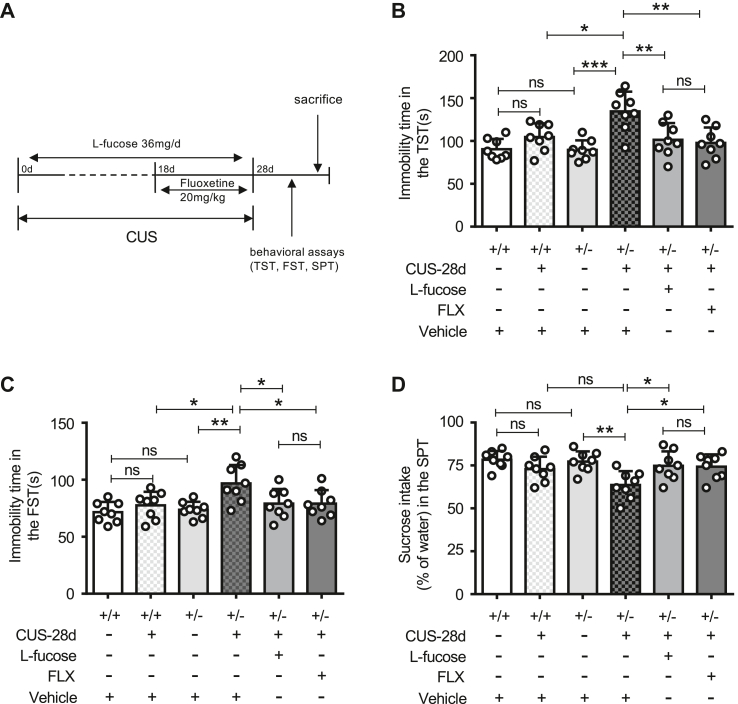
**L-fucose administration abolished depressive-like behaviors induced by CUS in *Fut8***^**+/−**^**mice.***A*, schedule of CUS modeling, with *Fut8*^+/−^ mice administered 36 mg/day of L-fucose or 20 mg/kg of fluoxetine (FLX) during the last 10 days. Behavioral tests were performed, and brain tissues were extracted afterward. *B*, the immobility time of mice in the TST. *C*, the immobility time of mice in the FST. *D*, the sucrose consumption of mice in the SPT. Data was analyzed by one-way ANOVA with Tukey's multiple comparison tests and shown as mean ± SD. n = 8 mice per group. n.s. *p* > 0.05; ∗*p* < 0.05; ∗∗*p* < 0.01; ∗∗∗*p* < 0.001.

Previous studies found that L-fucose has an inhibitory effect on LPS-induced neuroinflammation (45). Thus, we investigated whether exogenous L-fucose could affect the depressive-like behavior of *Fut8*^+/−^ mice subjected to CUS. The role of serotonin in CNS immunity is still unclear, but serotonin is indeed linked to inflammation (53, 54). The treatment of MDD patients with selective serotonin reuptake inhibitors (SSRIs) reduces IL-6 and TNF-α levels, and their antidepressant therapeutic effects may be partly attributed to their anti-inflammatory properties (55). The classic antidepressant FLX is an SSRI medication and is known to exert significant anti-neuroinflammatory effects (56), which is achieved by inhibiting microglia activation and reducing the release of inflammatory factors (57, 58, 59). FLX is often served as a positive control in studies of depression models (60, 61). Here, we also used FLX as a positive control to evaluate the degree of antidepressant effect of L-fucose since FLX is one of the most widely used drugs in clinical practice and has significant anti-inflammatory effects. As depicted in the schematic (Fig. 2*A*), L-fucose was administered intragastrically at a dose of 36 mg/day throughout the CUS model (45), while FLX was administered intraperitoneally at 20 mg/kg (62) during the last 10 days. Behavioral tests revealed that L-fucose eliminated the depressive-like behaviors in *Fut8*^+/−^ mice following CUS, exhibiting similar effects to FLX (Fig. 2). Exogenous L-fucose significantly decreased the immobility time in TST (Fig. 2*B*) and FST (Fig. 2*C*) and increased sucrose intake in the SPT (Fig. 2*D*). These results suggest that L-fucose may possess antidepressant effects similar to FLX.

### L-fucose suppression increased neuroinflammation induced by CUS in *Fut8*^+/−^ mice

**Figure 3:**
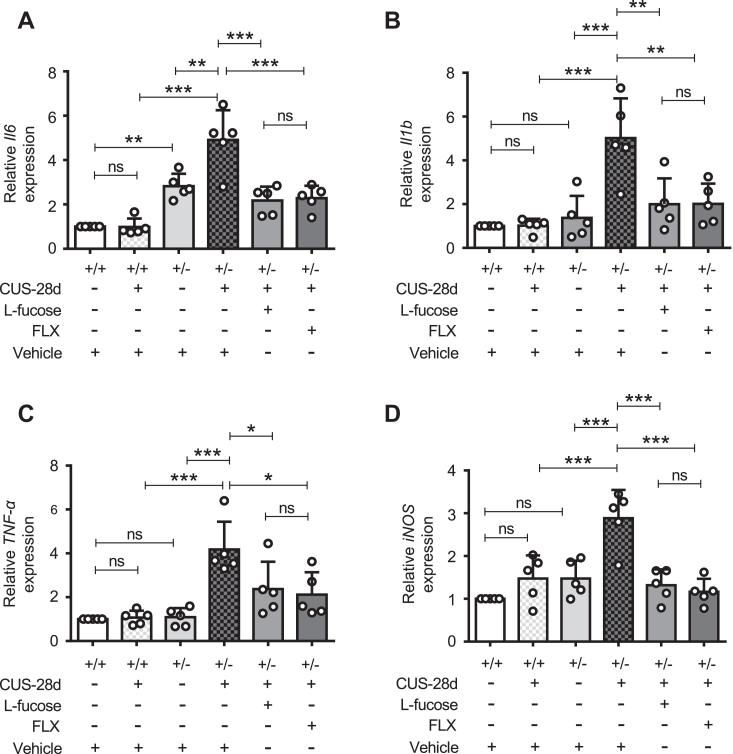
**L-fucose suppressed neuroinflammation induced by CUS in *Fut8***^**+/−**^**mice.** The mRNA levels of neuroinflammatory factors were analyzed by real-time PCR, including interleukin-6 (*Il6*) (*A*), interleukin 1 beta (*Il1b*) (*B*), tumor necrosis factor-alpha (*TNF-α*) (*C*), and inducible nitric oxide synthase (*iNOS*) (*D*) in the hippocampus. Glyceraldehyde-3-phosphate dehydrogenase (*Gapdh*) was used as an internal control. Values were normalized to that of the *Gapdh*. The value for *Fut8*^+/+^ mice with vehicle treatment was set as 1.0. The one-way ANOVA test with Tukey's multiple comparison test was used to calculate the quantitative data, which were displayed as the mean ± SD of five independent experiments. n = 5 mice per group. n.s. *p* > 0.05; ∗*p* < 0.05; ∗∗*p* < 0.01; ∗∗∗*p* < 0.001.

Neuroinflammation is implicated in MDD through various neurobiological mechanisms (7). Evidence indicates that CUS stress induces the release of pro-inflammatory cytokines, such as IL-6, IL-1β, TNF-α, and inducible NO synthase (iNOS) (18, 19, 20). The structure and function of the hippocampus have been linked to the pathophysiology and progression of MDD (63, 64, 65). Thus, we focused on the hippocampus to evaluate the effect of L-fucose on CUS-induced neuroinflammation. After 28 days of CUS, real-time PCR analysis revealed that the mRNA levels of the *Il6* (Fig. 3*A*), *Il1b* (Fig. 3*B*), *TNF-α* (Fig. 3*C*), and *iNOS* (Fig. 3*D*) were increased significantly in *Fut8*^+/−^ mice, but not in *Fut8*^+/+^ mice. Moreover, *Fut8*^+/−^ mice showed higher levels of *Il1b* mRNA than *Fut8*^+/+^ mice in the absence of CUS exposure. Treatment with exogenous L-fucose and FLX both obviously reversed the increased neuroinflammation in *Fut8*^+/−^ mice after CUS exposure. These findings suggest that L-fucose may reduce depressive-like behavior by mitigating neuroinflammation.

### L-fucose inhibited CUS-induced microglial activation in *Fut8*^+/−^ mice

**Figure 4:**
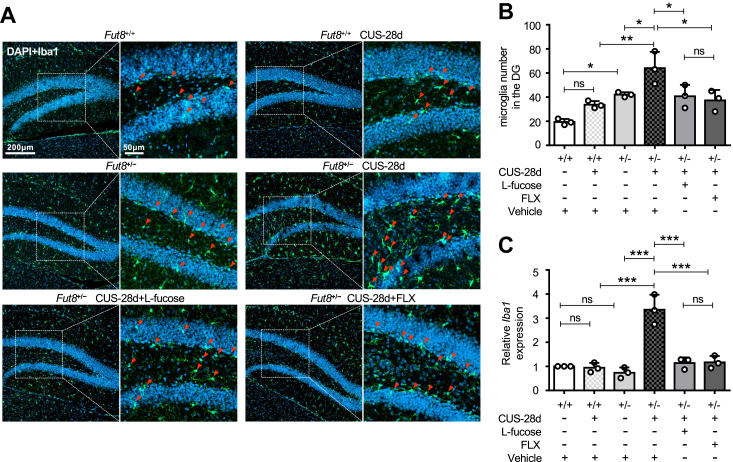
**L-fucose inhibited CUS-induced microglial activation.***A*, representative immunofluorescence staining images of the dentate gyrus (DG) in hippocampal coronal sections, showing Iba1 as a microglia marker. DAPI was used for nuclear staining. *Green* fluorescent cells co-localized with DAPI (*blue*) are identified as Iba1-positive microglia. Arrows indicate Iba1-positive cells. *B*, quantification of Iba1-positive microglia in the DG region using ImageJ software. The one-way ANOVA test with Tukey's multiple comparison test was used to calculate the quantitative data. Data from three independent experiments are presented as mean ± SD. n = 3 mice per group. n.s. *p* > 0.05; ∗*p* < 0.05; ∗∗*p* < 0.01. *C*, real-time PCR analysis of *Iba1* mRNA expression, normalized to *Gapdh* (internal control), and *Fut8*^+/+^ mice with vehicle treatment were set as 1.0. All data were analyzed by one-way ANOVA test with Tukey's multiple comparison test and displayed as the mean ± SD from three independent experiments. n = 3 mice per group. n.s. *p* > 0.05; ∗∗∗*p* < 0.001.

Microglia are innate immune cells of the CNS, and the alterations of number and function in microglia are closely related to the onset of depression (66, 67). It is widely known that chronic stress can induce the activation of microglia in specific brain regions and alter their phenotypic and functional properties (68). We performed immunofluorescence staining to detect the level of ionized calcium-binding adaptor molecule 1 (Iba1), a microglial activation marker in the dentate gyrus (DG) of the hippocampus (63, 69, 70, 71). After 28 days of CUS, the number of microglia in *Fut8*^+/−^ mice was significantly increased, which was suppressed after treatment with L-fucose and FLX (Fig. 4, *A* and *B*). Real-time PCR analysis also showed that the mRNA level of *Iba1* was significantly elevated in *Fut8*^+/−^ mice exposed to CUS, but not in *Fut8*^+/+^ mice. In addition, L-fucose or FLX exerted an effective suppression on this increase (Fig. 4*C*). Without CUS exposure, *Fut8*^+/−^ mice had more Iba1-positive cells in immunofluorescence results than *Fut8*^+/+^ mice (Fig. 4*B*); although there was no significant difference in the mRNA levels of *Iba1* between the two mice (Fig. 4*C*). These results further support the notion that core fucosylation negatively regulates microglia activation and consequently suppresses neuroinflammation.

### L-fucose attenuated the JAK2/STAT3 signaling pathway

**Figure 5:**
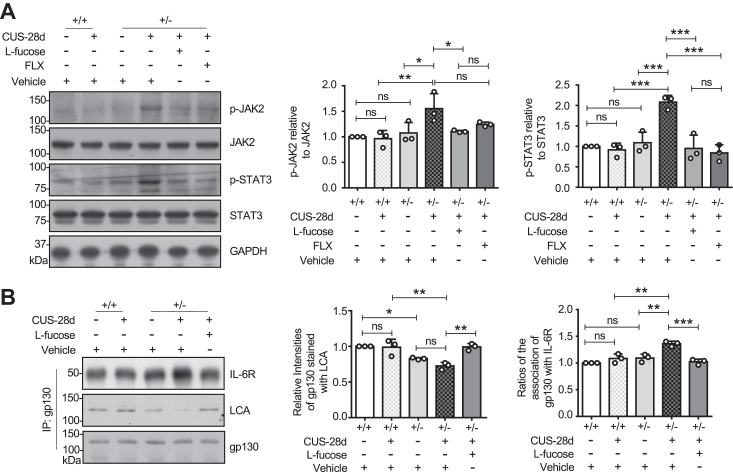
**L-fucose attenuated the JAK2/STAT3 signaling pathway.***A*, Western blot analysis of hippocampal tissues after 28 days of CUS, using antibodies against JAK2, phosphor-JAK2 (p-JAK2), STAT3, and phosphor-STAT3 (p-STAT3). GAPDH was used as a loading control. Data was quantified by ImageJ software. The ratio of p-JAK2 to JAK2 for *Fut8*^+/+^ mice treated with vehicle was set as 1.0. The ratio of p-STAT3 to STAT3 for *Fut8*^+/+^ mice treated with vehicle was set as 1.0. All data were analyzed by one-way ANOVA test with Tukey's multiple comparison test and displayed as the mean ± SD from three independent experiments. n = 3 mice per group. n.s. *p* > 0.05; ∗*p* < 0.05; ∗∗*p* < 0.01, ∗∗∗*p* < 0.001. *B*, equal amounts of hippocampus lysates were immunoprecipitated using an anti-gp130 antibody following a 28-days exposure to CUS. The immunoprecipitates were analyzed by western blotting with anti-IL-6R and anti-gp130 antibodies or lectin blotting with LCA lectin. ImageJ software was used to quantify the data. The ratio of LCA or IL-6R *versus* gp130 of *Fut8*^+/+^ mice treated with the vehicle was 1.0. All Data were shown as the mean ± SD from three independent experiments. n = 3 mice per group. n.s. *p* > 0.05; ∗*p* < 0.05; ∗∗*p* < 0.01, ∗∗∗*p* < 0.001. (One-way ANOVA test with Tukey's multiple comparison test).

The dysregulation of the JAK/STAT (Janus kinase-signal transducer and activator of transcription) signaling pathway plays a significant role in neuroinflammatory diseases (72, 73, 74). STAT3, a downstream effector of IL-6, is involved in the pro-inflammatory activation of microglia and astrocytes in various neuroinflammatory contexts (75, 76, 77). The inhibition of the JAK2/STAT3 pathway has been shown to have anti-neuroinflammatory and neuroprotective effects (78, 79). Meanwhile, FLX attenuated the neuroinflammation by suppressing the activation of JAK/STAT3 signaling (80). Western blotting has been used to assess the phosphorylation levels of JAK2 and STAT3. Quantitative analysis revealed that the phosphorylation levels of JAK2 and STAT3 were significantly increased in *Fut8*^+/−^ mice after 28 days of CUS, but not in *Fut8*^+/+^ mice. L-fucose could inhibit the elevated phosphorylation levels of JAK2 and STAT3 in *Fut8*^+/−^ mice subjected to CUS. FLX also displayed a similar inhibitory trend regarding the phosphorylation levels of JAK2 and STAT3, although no significant differences were observed for p-JAK2 (Fig. 5*A*). IL-6 signaling includes the gp130 receptor and the JAK/STAT pathway (81). We used co-immunoprecipitation to explore the molecular mechanisms for the effect of exogenous L-fucose administration in the CUS model. According to immunoprecipitation results, the interaction between gp130 and IL-6R was increased upon exposure to CUS in *Fut8*^+/−^ mice, compared to without CUS. Concomitantly, gp130 in *Fut8*^+/−^ mice exposed to CUS contained less core fucosylation than in *Fut8*^+/+^ mice or *Fut8*^+/−^ mice treated with L-fucose (Fig. 5*B*). Moreover, exogenous L-fucose administration inhibited the association between gp130 and IL-6R (Fig. 5*B*), which is similar to the observation in LPS-induced neuroinflammation (45). We further investigated the effects of WP1066, a JAK2/STAT3 inhibitor (82), on neuroinflammation and depression-like behaviors induced by CUS. After the first injection on the fourth day of the CUS model, the intraperitoneal injection was carried out once every 3 days at 30 mg/kg for nine injections, based on dose usage in the previous study (83). The effects of WP1066 on the behavioral experiments of depression were quite similar to those of L-fucose (Fig. S2, *A*–*C*). The combined treatment of WP1066 and L-fucose did not observe additive effects. Furthermore, the impact of WP1066 on the expression of inflammatory factors and *Fut8* in CUS-induced depression also showed a similar tendency with L-fucose (Fig. S3, *A*–*E*). However, WP1066 did not significantly suppress *Il1b* and *iNOS* expression. The combined treatment of WP1066 and L-fucose did not observe additive effects, except for the phosphorylated JAK2 levels (Fig. S3*F*). These results further suggest the suppression of CUS-induced depression by exogenous L-fucose *via* neutralizing neuroinflammation. These results indicate that L-fucose exerts its anti-inflammatory effects by inhibiting the JAK2/STAT3 pathway, thereby playing an antidepressant role.

### Exogenous L-fucose reversed CUS-induced damage in synaptic plasticity

**Figure 6:**
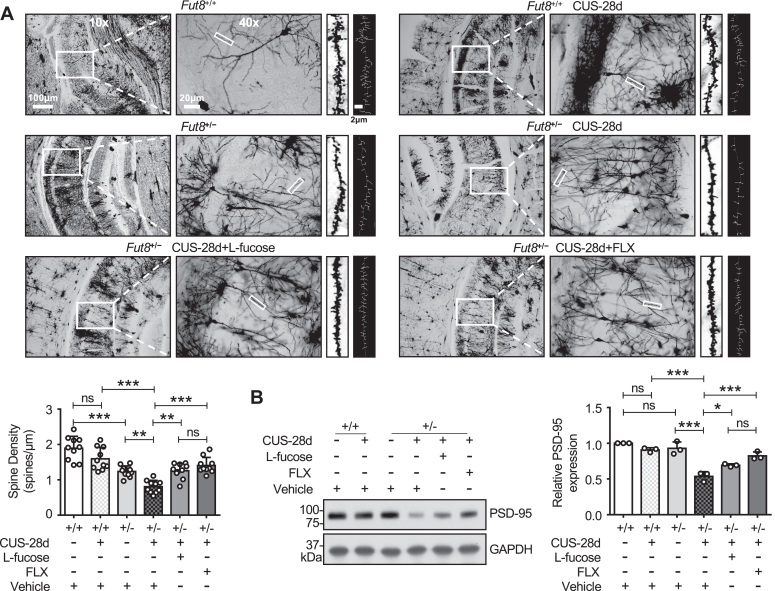
**Exogenous L-fucose reversed CUS-induced damage in synaptic plasticity.***A*, representative images of the dendritic spine in Golgi staining. Images of the hippocampal region under 10x and 40x magnifications, as well as representative synaptic spine images and ImageJ-processed images, are shown from *left* to *right*. Spine density is the number of spines divided by the dendritic shaft length. ImageJ software measured the number of branches and total dendritic length. Data from 10 dendritic segments across three mice is mean ± SD. n.s. *p* > 0.05; ∗∗*p* < 0.01; ∗∗∗*p* < 0.001 (one-way ANOVA test with Tukey's multiple comparison test). *B*, Western blotting analysis of PSD-95 levels in hippocampal tissues after 28 days of CUS, quantified using ImageJ. GAPDH was used as a loading control, and the ratio of PSD-95 to GAPDH for *Fut8*^+/+^ mice treated with vehicle was set as 1.0. All data were analyzed by one-way ANOVA test with Tukey's multiple comparison test and displayed as the mean ± SD from three independent experiments. n = 3 mice per group. n.s. *p* > 0.05; ∗*p* < 0.05; ∗∗∗*p* < 0.001.

Through the activation of microglia, inflammatory cytokine overproduction causes synaptic dysfunction and neuronal damage in the hippocampus, which in turn induces depressive behaviors (24). Spines possess highly dynamic, and the alterations in their density and morphology are the basis of structural synaptic plasticity (84). Neuroinflammation following depression may lead to impaired synaptic plasticity, decreased dendritic spine density, and synaptic plasticity-related proteins, such as postsynaptic density protein 95 (PSD-95) (47, 85). FLX has the effect of improving synaptic plasticity (86, 87). Given the observed behavioral alterations and cytokine levels in *Fut8*^+/−^ mice, we hypothesized that neuroinflammation triggered from excessive microglia activation may affect neuronal plasticity. To explore the role of L-fucose in synaptic plasticity, we need to detect the spine density through Golgi staining. Decreased dendritic spine density in the CA1 hippocampus region is associated with depressive-like behavior following CUS (88). Therefore, we examined the synaptic density in the CA1 region of the mouse hippocampus. The results demonstrated that synaptic density in the hippocampus of *Fut8*^+/−^ mice was lower than that in *Fut8*^+/+^ mice. There was no significant change in synaptic density in *Fut8*^+/+^ mice after 28 days of CUS (Fig. 6*A*). After 28 days of CUS, synaptic densities in *Fut8*^+/−^ mice were significantly decreased, whereas L-fucose and FLX significantly alleviated this impairment (Fig. 6*A*). Additionally, the level of synaptic plasticity-related proteins was detected by western blotting. We found the expression level of PSD-95 was significantly suppressed in the hippocampus of *Fut8*^+/−^ mice exposed to the CUS, compared to the control without CUS (Fig. 6*B*). Importantly, L-fucose or FLX partially rescued the decreased PSD-95 expression in *Fut8*^+/−^ CUS mice (Fig. 6*B*). These data suggest that L-fucose may exert antidepressant effects by restoring synaptic plasticity.

### L-fucose recovered the CUS-induced down-regulation of Fut8 expression

**Figure 7:**
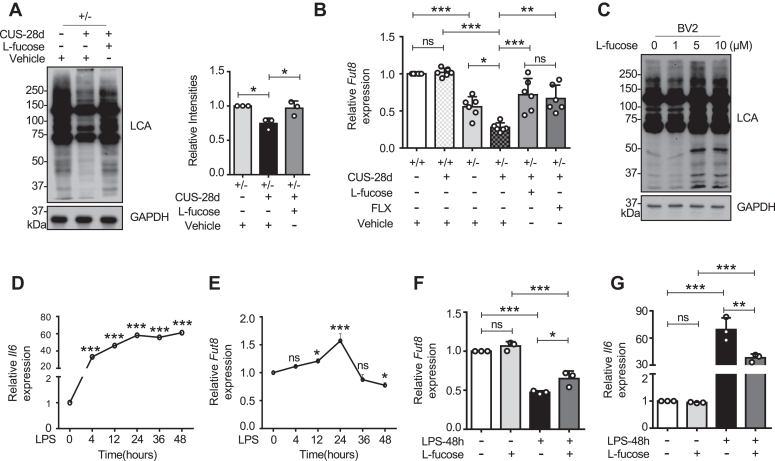
**L-fucose rescued the CUS-induced down-regulation of Fut8 expression.***A*, Lectin blot analysis of hippocampal tissues after 28 days of CUS, using Lens Culinaris Agglutinin (LCA), specifically recognizing Core fucosylation. GAPDH was used as a loading control. The ratio of LCA *versus* GAPDH of *Fut8*^+/−^ mice with the vehicle was set as 1.0. All data were analyzed by one-way ANOVA test with Tukey's multiple comparison test and displayed as the mean ± SD from three independent experiments. ∗*p* < 0.05. *B*, real-time PCR analysis of *Fut8* mRNA expression. *Gapdh* was used as an internal control. Each value was normalized to that of the *Gapdh*. The values of *Fut8 versus Gapdh* in *Fut8*^+/+^ mice treated with vehicle were set to 1.0. All data were analyzed by one-way ANOVA test with Tukey's multiple comparison test and displayed as the mean ± SD from six independent experiments. n = 6 mice per group. n.s. *p* > 0.05; ∗*p* < 0.05; ∗∗*p* < 0.01; ∗∗∗*p* < 0.001. *C*, BV2 cells were treated with L-fucose at the indicated concentrations and cultured for 48 h. The same amounts of cell lysates were lectin blotted with LCA. GAPDH served as a loading control. *D* and *E*, BV2 cells were treated with LPS at 1 μg/ml for the indicated times. Real-time PCR was used to analyze the mRNA levels of *Il6* and *Fut8*. *Gapdh* was used as an internal control. Each value was normalized to that of the *Gapdh*. *Il6* or *Fut8 versus Gapdh* values in BV2 cells without LPS treatment were set to 1.0. All data were analyzed by one-way ANOVA with Tukey's multiple comparison test and shown as the mean ± SD from three independent experiments. n.s. *p* > 0.05; ∗*p* < 0.05; ∗∗∗*p* < 0.001. *F* and *G*, after pretreatment with or without L-fucose at 5 μM for 4 h, the cells were stimulated with LPS for 48 h. Real-time PCR was used to analyze the mRNA levels of *Il6* and *Fut8*. *Gapdh* was used as an internal control. Each value was normalized to that of the *Gapdh*. *Il6* or *Fut8 versus Gapdh* values in BV2 cells without LPS and L-fucose treatment were set to 1.0. All data were analyzed by one-way ANOVA with Tukey's multiple comparison test and displayed as the mean ± SD from three independent experiments. n.s. *p* > 0.05; ∗*p* < 0.05; ∗∗*p* < 0.01; ∗∗∗*p* < 0.001.

To investigate the alteration in core fucosylation after CUS exposure, we checked the level of core fucosylation *via* lectin blotting using the Lens Culinaris Agglutinin (LCA). LCA can specifically recognize core fucosylation (89). After 28 days of CUS, *Fut8*^+/−^ mice exhibited a significant reduction in core fucosylation levels, which was reversed by exogenous L-fucose (Fig. 7*A*). Since L-fucose administration may also affect other fucosylation (90), and studies have found that mice lacking α1,3-fucosyltransferase IX exhibit increased anxiety-like behaviors (91), we also examined the expression levels of Lewis X by western blotting with anti-Lewis X antibody. After CUS exposure, curiously, both *Fut8*^+/+^ and *Fut8*^+/−^ mice exhibited decreased Lewis X expression levels, which could not be rescued by L-fucose treatment (Fig. S4, *A* and *B*), suggesting the effect of L-fucose on CUS is not dependent on expression levels of Lewis antigen. Given the notable increase in inflammatory factors in *Fut8*^+/−^ CUS mice, we hypothesized that inflammation might downregulate *Fut8* gene expression. Real-time PCR analysis of *Fut8* mRNA levels showed a significant decrease in *Fut8* expression due to CUS-induced inflammation in *Fut8*^+/−^ mice. However, this downregulation was reversed in *Fut8*^+/−^ CUS mice treated with L-fucose or FLX (Fig. 7*B*). These findings suggest that neuroinflammation may downregulate *Fut8* expression. To investigate if the suppression of inflammation or cell autonomy is responsible for the upregulation of *Fut8* mRNA following L-fucose treatment, we established the neuroinflammation model by treating BV2 cells cultured *in vitro* with LPS (1 μg/ml) (92). The results of LCA lectin blotting demonstrated that the core fucosylation levels were increased by L-fucose. The dose of 5 μM L-fucose was adequate for the increasing effect (Fig. 7*C*). After LPS treatment, *Il6* mRNA levels increased significantly (Fig. 7*D*), and *Fut8* mRNA levels increased after 12 h and 24 h of LPS treatment. Interestingly, as the LPS treatment time was postponed, *Fut8* mRNA levels decreased (Fig. 7*E*), suggesting bi-phase responses in the induction. BV2 cells were pretreated with L-fucose for 4 h and then stimulated with LPS for 48 h. The real-time PCR analysis showed decreased *Fut8* expression while increasing *Il6* expression, significantly rescued by L-fucose (Fig. 7, *F* and *G*). Curiously, L-fucose alone did not change *Fut8* expression (Fig. 7*F*).

### Effects of L-fucose on *Fut8*^+/+^ mice exposed to CUS

**Figure 8:**
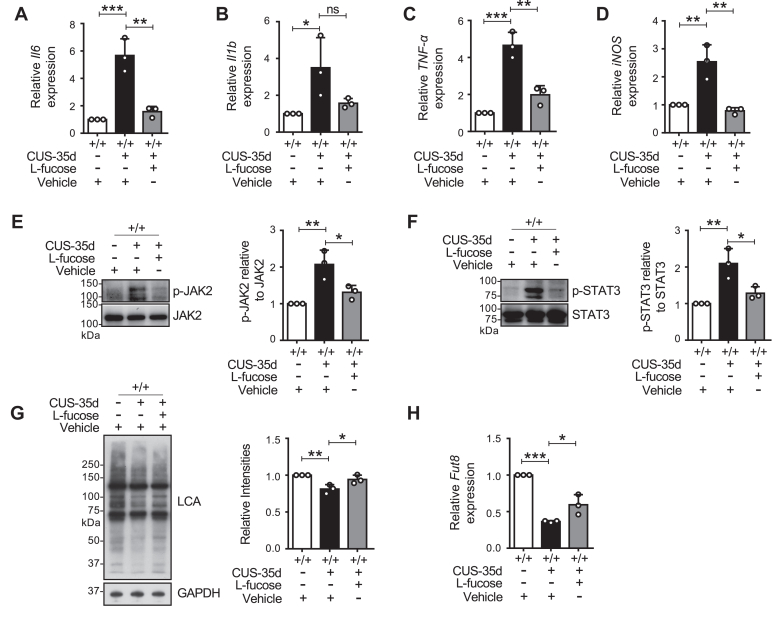
**Effects of L-fucose on *Fut8***^**+/+**^**mice exposed to CUS at 35 days.***Fut8*^+/+^ mice were subjected to the CUS for 35 days and treated with L-fucose. *A*–*D*, the mRNA levels of cytokines were analyzed by real-time PCR, including *Il6* (*A*), *Il1b* (*B*), *TNF-α* (*C*), and *iNOS* (*D*) in the hippocampus. The *Gapdh* was used to normalize each value. The value of *Il6*, *Il1b*, *TNF-α* or *iNOS versus Gapdh* in *Fut8*^+/+^ mice treated with the vehicle was set as 1.0. All data were analyzed by one-way ANOVA test with Tukey's multiple comparison test and displayed as the mean ± SD from three independent experiments. n = 3 mice per group. n.s. *p* > 0.05; ∗*p* < 0.05; ∗∗*p* < 0.01; ∗∗∗*p* < 0.001. *E* and *F*, Western blotting analyses for p-JAK2 (*E*) and p-STAT3 (*F*) were conducted on hippocampal tissues, with data quantified using ImageJ software. The ratios of p-JAK2 to JAK2 and p-STAT3 to STAT3 for *Fut8*^+/+^ vehicle-treated mice were set as 1.0. GAPDH was used as a loading control. All data were analyzed by one-way ANOVA test with Tukey's multiple comparison test and displayed as the mean ± SD from three independent experiments. n = 3 mice per group. ∗*p* < 0.05; ∗∗*p* < 0.01. *G*, lectin blot analysis with LCA, normalized to GAPDH, with the *Fut8*^+/+^ vehicle-treated mice set as 1.0. Data were shown as mean ± SD from three independent experiments. n = 3 mice per group. ∗*p* < 0.05; ∗∗*p* < 0.01. (One-way ANOVA test with Tukey's multiple comparison test). *H*, real-time PCR analysis of *Fut8* mRNA level. *Gapdh* was used as a loading control. The *Gapdh* was used to normalize each value. The value of *Fut8 versus Gapdh* in *Fut8*^+/+^ vehicle-treated mice set as 1.0. Data are shown as mean ± SD from three independent experiments. n = 3 mice per group. ∗*p* < 0.05; ∗∗∗*p* < 0.001. (One-way ANOVA test with Tukey's multiple comparison test).

We examined the effects of L-fucose on *Fut8*^+/+^ mice after CUS exposure to further explore the role of L-fucose in *Fut8*^+/+^ mice. Following 35 days of CUS, *Fut8*^+/+^ mice displayed increased immobility time in TST (Fig. S5*A*) and FST (Fig. S5*B*), alongside reduced sucrose preference in the SPT (Fig. S5*C*) compared to the controls without CUS. Importantly, these alterations were ameliorated by L-fucose treatment. Additionally, the real-time PCR analysis showed that the elevated mRNA levels of *Il6* (Fig. 8*A*), *Il1b* (Fig. 8*B*), *TNF-α* (Fig. 8*C*), and *iNOS* (Fig. 8*D*) in *Fut8*^+/+^ mice following CUS were significantly suppressed by exogenous L-fucose. The levels of phospho-JAK2 (Fig. 8*E*) and phospho-STAT3 (Fig. 8*F*) were also significantly increased in *Fut8*^+/+^ mice after CUS, which were inhibited by L-fucose treatment. Measurements of core fucosylation using LCA revealed that CUS exposure resulted in a significant decrease in LCA reactivity, which was partially rescued by L-fucose (Fig. 8*G*). Notably, *Fut8* mRNA expression levels were downregulated in *Fut8*^+/+^ mice after CUS (Fig. 8*H*), and this downregulation could also be partially reversed by L-fucose treatment. These results strongly indicate that core fucosylation is crucial for CNS maintenance, and L-fucose treatment can mitigate depressive-like behaviors in *Fut8*^+/+^ mice.

## Discussion

**Figure 9:**
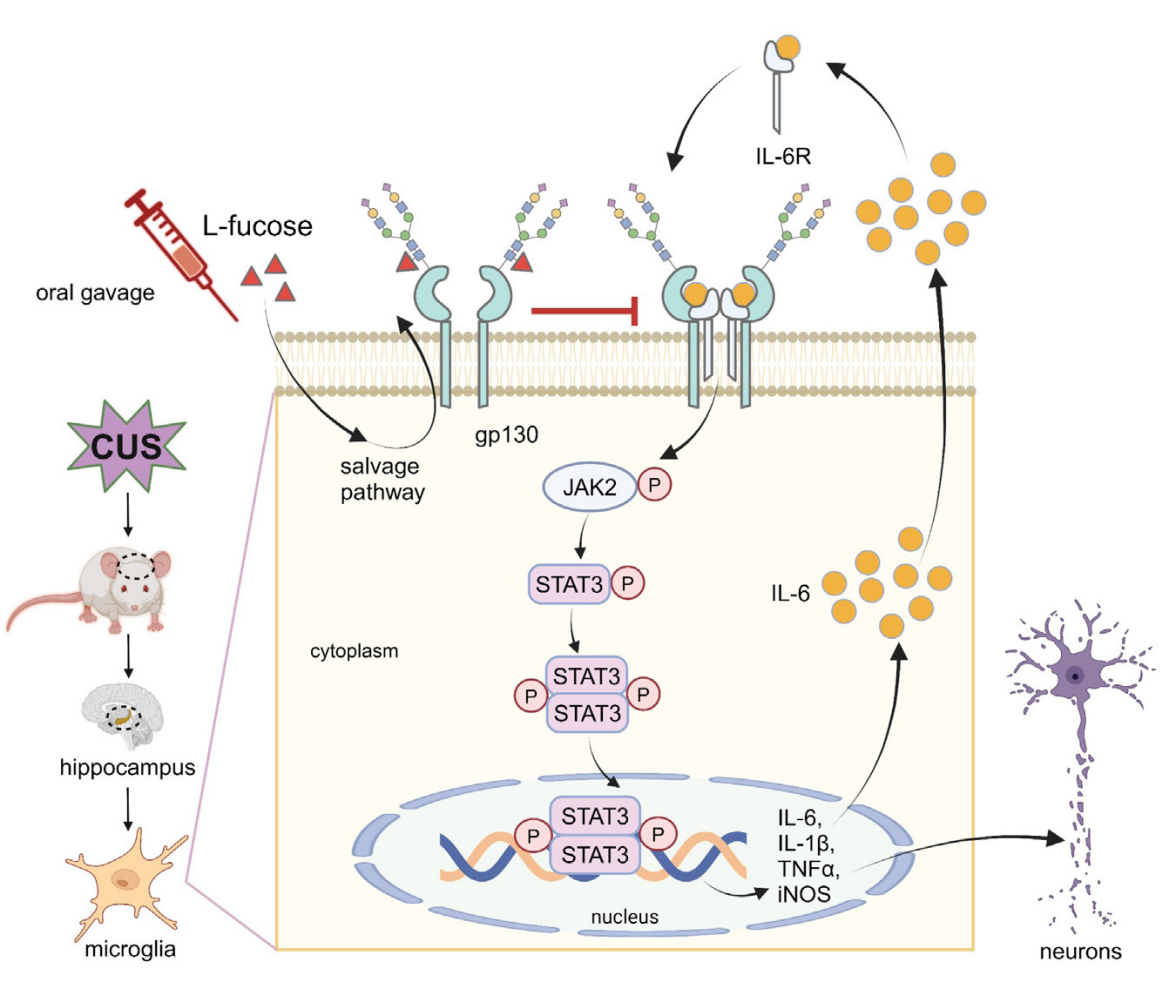
**L-fucose inhibits neuroinflammation and acts as an antidepressant.***Fut8*^+/−^ mice exhibited depressive-like behaviors earlier than *Fut8*^+/+^ mice following CUS and displayed excessive microglia activation along with elevated cytokine expression levels. These changes may impair neuronal synaptic plasticity. Administration of exogenous L-fucose enhanced core fucosylation of gp130 through the salvage pathway for GDP-fucose synthesis, subsequently preventing the complex formation between gp130 and IL-6R, then inhibiting hippocampal microglia activation and subsequent neuroinflammation *via* the JAK2/STAT3 pathway. This intervention may reverse the synaptic plasticity disorder induced by CUS and ultimately alleviate depressive-like behaviors. Our previous research showed that the suppressive effect of L-fucose on IL-6 induction by LPS was observed only in the *Fut8*^+/+^ cells, not in the *Fut8*^−/−^ microglia (45), highlighting the significance of core fucosylation in neuroinflammation. Additionally, complex formation between gp130 and IL-6R was significantly increased in the *Fut8*^−/−^ cells compared to *Fut8*^+/+^ cells. Thus, we conclude that L-fucose inhibits neuroinflammation and acts as an antidepressant, at least partly through the upregulation of core fucosylation, which negatively regulates specific cytokine receptors, including the interaction between gp130 and IL-6R. This figure is created with BioRender.com.

Our current study demonstrated that excessive microglia activation caused *Fut8*^+/−^ mice to exhibit depressive-like behaviors and increased neuroinflammatory factors earlier than *Fut8*^+/+^ mice following exposure to CUS. However, increased core fucosylation through exogenous L-fucose inhibited the overactivation of hippocampal microglia *via* the JAK2/STAT3 pathway, suppressed subsequent hippocampal neuroinflammation, reversed CUS-induced impairments in synaptic plasticity, and ultimately abolished the depressive-like behavior in *Fut8*^+/−^ mice (Fig. 9).

Changes in microglia activity are associated with the onset of depression (21, 22). Multiple studies have reported an increase in microglia numbers in the hippocampus of animal models of depression exposed to chronic stress (93, 94, 95, 96). It has also been shown that the density of glial cells in the dentate gyrus of the hippocampus is significantly increased in MDD (97). Our results indicate that the total number of Iba1-positive microglia in the hippocampus DG area of *Fut8*^+/−^ mice after 28 days of CUS was significantly higher than that in control mice, suggesting that CUS exposure increases microglia numbers, which is suppressed by L-fucose administration. Numerous studies highlight the central role of neuroinflammation in the development of MDD (14, 15). Stress can cause homeostasis dysregulation, resulting in excessive microglia activation, and cause the production of proinflammatory cytokines. This neuroinflammation impairs neurons subsequently and contributes to depressive symptoms (25, 26). This study focused only on microglia. That does not mean that other cells, such as astrocytes and neurons, are not involved in neuroinflammation because core fucosylation is highly expressed in brain tissue (42). Depression is also known to be associated with astrocyte-mediated neuroinflammation (98). This study mainly focused on the relationship between IL-6/gp130-mediated signaling, a key inflammatory response pathway, and core fucosylation, while the IL-6R is present in microglia but not in astrocytes (99). In addition to the neuroinflammation hypothesis, there are other hypotheses about depression, such as the monoamine hypothesis, hypothalamic-pituitary-adrenal axis dysfunction hypothesis, *etc.* (7). In subsequent studies, we will use new technologies to explore whether other cells or other mechanisms are involved in the relationship between core fucosylation and depression.

Several studies have reported that the levels of multiple inflammatory markers were increased in patients with MDD (15, 16). Notably, alterations in IL-6 serum levels are among the most reproducible abnormalities in depression patients (16). Children with high circulating IL-6 levels have a 10% higher risk of developing MDD in adulthood compared to the general population or children with low IL-6 levels (100). Furthermore, it has been reported that two-thirds of mice exhibit depressive-like behaviors after exposure to social defeat stress (101). In our study, we found that *Fut8*^+/−^ mice had higher *Il6* levels than the *Fut8*^+/+^ group (Fig. 3*A*), indicating that diminished core fucosylation predisposes mice to depression (Fig. 2). Additionally, other studies also suggest a link between core fucosylation and neuroinflammation in the central nervous system (CNS). For instance, core-fucosylated *N*-glycans were significantly decreased in mouse brain tissues treated with LPS (102). FUT8 expression was also significantly reduced in schizophrenia patients compared to controls (103). Patients with Multiple sclerosis exhibited lower levels of core-fucosylated *N*-glycans compared to matched controls (104), a similar finding also observed in Alzheimer's disease (105). Interestingly, it has been reported that female patients with MDD show decreased core fucosylation and increased IL-6 levels in serum (106). Consistent with these studies, we observed decreased core fucosylation levels in the hippocampus of mice exposed to the CUS model (Figs. 7*A*, 8*G*), which was supported by the observation in the BV2 cell model treated with LPS (Fig. 7*F*). L-fucose treatment of BV2 cells alone did not cause changes in *Fut8* mRNA levels. However, L-fucose could reduce the LPS-induced inflammation level in BV2 cells, thereby affecting the *Fut8* mRNA level, indicating that inflammation is the cause of alterations in *Fut8* mRNA levels (Fig. 7, *F* and *G*). It is notable to mention that there are bi-phase responses in LPS induction. The mRNA levels of *Fut8* were significantly increased during the shorter time treatment, such as 12 and 24 h, but decreased during the longer time treatment (Fig. 7*E*). Thus, the phenomenon could be explained by the possibility that *Fut8* mRNA levels are upregulated in response to acute inflammatory stimulation and downregulated in response to chronic inflammatory stimulation. This is consistent with the findings that our chronic depression model CUS stimulation mice exhibit reduced *Fut8* mRNA levels (Figs. 7*B*; 8*H*). The underlying mechanism for L-fucose-upregulating *Fut8* has yet to be entirely understood. However, based on the observations in (Fig. 7, *D*–*F*), we speculate that chronic inflammation may suppress *Fut8* expression, while L-fucose can reduce chronic inflammation, which results in upregulation of *Fut8*. On the other hand, acute inflammation induces the mRNA level of *Fut8* (Fig. 7*E*). The underlying mechanism for the different responses to acute and chronic inflammation remains to be studied further. These above results further suggest that core fucosylation is important for CNS maintenance and negatively regulates neuroinflammation.

The reduction or loss of core fucosylation may upregulate the pro-inflammatory reaction. Previous research indicated that a lack of core fucosylation leads to emphysema-like changes in *Fut8*^−/−^ mice (107). Moreover, in a cigarette smoke-induced emphysema model, *Fut8*^+/−^ mice were more sensitive than *Fut8*^+/+^ mice (108). After LPS treatment, IL-6 expression levels in the lung and brain tissues of *Fut8*^+/−^ mice were significantly higher than in *Fut8*^+/+^ mice, and L-fucose treatment could reduce the IL-6 levels in *Fut8*^+/−^ mice (45, 108). The GDP-fucose level in *Fut8*^+/−^ mice was lower than that in *Fut8*^+/+^ mice, and exogenous administration of L-fucose could significantly increase the GDP-fucose level in *Fut8*^+/−^ mice (45). Therefore, exogenous L-fucose administration can increase core fucosylation in *Fut8*^+/−^ mice. Mechanistic studies suggested that core fucosylation negatively regulates the formation of IL-6R and gp130 complex by increasing core fucosylation of gp130 in *Fut8*^+/−^ mice, this complex can mediate the JAK2/STAT3 pathway (Fig. 5). Similarly, we found that after CUS stress exposure, neuroinflammatory factors, including IL-6, were significantly higher in *Fut8*^+/−^ mice compared to *Fut8*^+/+^ mice, while exogenous L-fucose significantly inhibited their expression. It is noteworthy that GDP-fucose from exogenous L-fucose is preferentially utilized by Fut8 (109). This may explain why the expression levels of Lewis X were not increased in mice exposed to CUS with L-fucose treatment (Fig. S4, *A* and *B*). Although core fucosylation and other fucosylation, such as α1,3-fucosylation, were reduced after CUS, core fucosylation, not α1,3-fucosylation on Lewis X, could partially be rescued by L-fucose (Figs. 7*A*; 8*G*). Our previous study also showed that administration of exogenous L-fucose rescued the reduced IgG amount in *Fut8*^+/−^ mice (110). We believe that the effects of L-fucose on CUS-induced depression are Fut8-dependent, as a previous study indicated that supplementation of L-fucose suppressed the enhanced IL-6 expression level in *Fut8*^+/+^ cells, but not in *Fut8*^−/−^ cells (45). We do not exclude other potential functions of L-fucose, which warrant further investigation. It has been reported that serotonin release is reduced in patients with depression (111). Lower levels of serotonin release in key parts of the brain may lead to stronger suicidal thoughts and higher mortality rates in people with depression (112). In our previous study, we observed the levels of serotonin metabolites were reduced in the *Fut8*^*−/−*^ mice compared with *Fut8*^*+/+*^ mice (42), suggesting that core fucosylation may be associated with serotonin metabolism. Furthermore, in this experiment, we found that the effect of L-fucose was similar to that of FLX. Perhaps L-fucose administration can increase the level of serotonin in the brain and thus alleviate the depressive-like behavior caused by CUS. We will conduct further exploration on this aspect in the future. Taken together, these data suggest that core fucosylation negatively regulates inflammation induced by CUS exposure.

One of the key contributors to refractory depression is the dysregulation of the JAK2/STAT3 signaling pathway (74). In the nervous system, the JAK-STAT pathway plays a crucial role in neurons, astrocytes, and oligodendrocytes (113, 114, 115). This pathway is also critical in the complex relationship between depression and systemic manifestations of chronic inflammation (116, 117). Inhibition of STAT3 can block IL-6 effects *in vitro* and regulate depressive-like behaviors *in vivo* (118). Microglial STAT3 inhibition may improve depressive behavior through neuron-microglia interaction (119). These findings suggest that targeting this pathway could enhance existing depression treatments. In our study, we found that L-fucose administration inhibited the JAK2/STAT3 signaling pathway and reduced neuroinflammation in microglia induced by CUS exposure, indicating that L-fucose can target the JAK2/STAT3 pathway to exert its antidepressant effect. It is notable that although there was no significant difference between L-fucose and WP1066, JAK2/STAT3 inhibitor, in their antidepressant effects (Fig. S2, *A*–*C*), the combined administration of L-fucose and WP1066 had a better inhibitory effect on p-JAK2 than L-fucose alone (Fig. S3*F*). This may indicate that L-fucose exerts anti-inflammatory and antidepressant effects through not only the JAK2/STAT3 pathway but also other signal pathways. And this point needs further exploration.

Higher depression severity has been linked to lower synaptic density in the hippocampus, dorsolateral prefrontal cortex, and anterior cingulate cortex in imaging synaptic density in depressed patients (120). Strong inflammatory activation disrupts the normal microglia structure and function, contributing to depression and neuroplasticity impairment (24). Chronic stress, such as CUS, inhibits the expression of synaptic proteins, including PSD-95, synaptophysin, and decreases dendritic spine density (47, 121, 122). Our study found that the synaptic density in *Fut8*^+/−^ mice exposed to CUS was significantly reduced in the hippocampus, and the protein expression of PSD-95 was also diminished, likely due to the release of inflammatory factors by microglia, which disrupts synaptic plasticity (Fig. 6, *A* and *B*). We also found that synaptic density in *Fut8*^*+/−*^ mice was lower than that in *Fut8*^*+/+*^ mice (Fig. 6*A*); this suggests that decreased core fucosylation may affect synaptic density in mice. JAK2 and STAT3 are highly expressed in the brain and are involved in synaptic plasticity (123). Inhibition of neuroinflammation caused by spinal cord injury through the JAK2/STAT3 signaling pathway can prevent neuronal apoptosis (124). IL-6 inhibits LTP, and blocking endogenous IL-6 can enhance LTP duration and long-term memory while reducing neuronal damage (125, 126). The absence of the *Fut8* gene significantly suppressed LTP in *Fut8*^−/−^ mice (43). Interestingly, 30 years ago, Matthies *et al.* reported that L-fucose and fucosyllactose could enhance hippocampal LTP *in vitro* (127), although the underlying mechanism was unclear. This study provides a molecular mechanism by which exogenous L-fucose increases core fucosylation of gp130, negatively regulating the JAK2/STAT3 signaling pathway to ameliorate neuroinflammation of CUS maintenance (Figs. 5*B*; 9).

In summary, L-fucose negatively regulates CUS-induced neuroinflammation and improves synaptic plasticity impairment through the JAK2/STAT3 pathway, ultimately exerting an antidepressant effect, indicating that L-fucose possesses significant anti-neuroinflammatory activity and antidepressant potential.

## Experimental procedures

### Antibodies and reagents

Biotinylated LCA (J207) was obtained from J-oil Mills. The anti-GAPDH antibody (G9545) and anti-Mouse IgG antibody (AP124P) were obtained from Sigma. Anti-JAK2 (catalog no.: 3230), anti-Phospho-JAK2 (catalog no.: 3771), anti-STAT3 (catalog no.: 9139), anti-Phospho-STAT3 (catalog no.: 9145) antibodies and anti-rabbit IgG, HRP-linked antibody (catalog no.: 7074) were acquired from Cell Signaling Technology. The Iba1 antibody (catalog no. 019–19741) was purchased from FUJIFILM Wako Pure Chemical Corporation, and the anti-PSD-95 antibody (catalog no.:04–1066) was obtained from Millipore Corporation. The ABC kit (catalog no.: PK-4000) was from Vector Laboratories, and the goat anti-rabbit antibody Alexa Fluor 488 (catalog no.: A-11008) was obtained from Invitrogen. L-fucose (F0065) and Anti-Lewis X (A2578) were acquired from TCI, while fluoxetine (56,296–78–7) was sourced from Merck. WP1066 (HY-15312) was acquired from MCE. LPS was obtained from Sigma and purified from *Escherichia coli* O111:B4 (L2630). Ab-Capcher MAG2 was from ProteNova. The anti-IL-6R (#MA5-29721) and anti-gp130 (#A304–929A) antibodies were purchased from Thermo Fisher Scientific.

### Animal

*Fut8*^+/−^ heterozygous mice with the ICR genetic background were intercrossed to generate *Fut8*^+/+^ littermates and *Fut8*^+/−^ mice (42). To investigate the underlying mechanism, we used a sensitive inflammation-monitoring mouse system containing the human interleukin-6 (*hIL6*) bacterial artificial chromosome (BAC) transgene modified with luciferase (*Luc*) reporter cassette (128). The *Fut8*^+/−^ mice were mated with *hIL6*-BAC-*Luc* reporter transgenic mice to produce *Fut8*^+/+^::*hIL6*-*Luc* and *Fut8*^+/−^::*hIL6*-*Luc* compound transgenic mice for LPS-induced inflammation as described (45). All experiments were conducted using 5- to 6-week-old male mice weighing approximately 25 to 30 g. Mice were housed in groups under standard vivarium conditions, with free access to food and water, a 12-h light-dark cycle (lights on from 7:00–19:00), an ambient temperature of 22 ± 2 °C, and relative humidity of 55 ± 5%. Mice received oral L-fucose twice daily *via* gavage at a dose of 36 mg/day. All animal experiments adhered to protocols approved by the Animal Care and Use Committee of the Graduate School of Pharmaceutical Sciences, Tohoku Medical and Pharmaceutical University.

### Cell culture

BV2 (mouse microglia cell line) was generously gifted by Professor Elisabetta Blasi (University of Modena and Reggio Emilia, Modena). At 37 °C and 5% CO2, BV2 cells were cultured in Dulbecco's modified Eagle's medium supplemented with 10% fetal bovine serum in a humidified environment. The cells were confirmed free from *mycoplasma* using the e-Myco *Mycoplasma* PCR Detection kit (iNtRON Biotechnology, Republic of Korea).

### Chronic unpredictable stress (CUS)

Following established procedures (62), we constructed the CUS model by randomly exposing mice to two stressors daily for 28 days (*Fut8*^+/−^ mice) or 35 days (*Fut8*^+/+^ mice), as detailed in Table 1. Stressors included 1 h of cage shaking, 12 h of lights-on during the night, 3 h of lights-off during the day, 2 h of light restraint (housing in small cages), 1 h of restraint (placing mice in a 50 ml plastic tube with breathing holes), 14 h of 45°cage tilt, 14 h of wet cage, and 12 h of food or water deprivation during the dark period.

**Table 1:** Construction of the CUS model, in which mice were randomly exposed to two stressors daily

Times	Monday	Tuesday	Wednesday	Thursday	Friday	Saturday	Sunday
Week 1	lights-off cage tilt	shaking lights-on	no water no food	restraint lights-on	light restraint wet cage	lights-off lights-on	shaking cage tilt
Week 2	restraint no water	light restraint no food	lights-off lights-on	restraint shaking	lights-off cage tilt	shaking wet cage	light restraint no water
Week 3	lights-off cage tilt	light restraint lights-on	restraint no food	shaking cage tilt	no water wet cage	lights-off no food	light restraint lights-on
Week 4	restraint no water	lights-off cage tilt	shaking wet cage	light restraint lights-on	restraint no food	shaking wet cage	lights-off cage tilt
Week 5	shaking no food	lights-off lights-on	restraint cage tilt	shaking no water	light restraint wet cage	lights-off no food	shaking cage tilt

### Tail suspension test (TST)

The TST was performed as previously described (62). Mice were suspended 50 cm above the ground with tape attached to their tails (1 cm from the tip) for 6 min. A blinded researcher recorded the duration of immobility during the last 4 min. Mice were considered immobile only when they were passively suspended and completely motionless. Any mice that climbed up the tail were excluded from the analysis.

### Forced swimming test (FST)

The FST was conducted as previously outlined (62). Mice were placed individually in a transparent glass cylinder (height: 25 cm, diameter: 10 cm) filled with 10 cm of water at 25 ± 1 °C for 6 min. An investigator unaware of the study recorded the immobility time during the last 4 min, defined as the duration the mouse floated in the water without struggling, only making movements necessary to keep its head above water.

### Sucrose preference test (SPT)

This experiment followed established protocols (62). Mice were given access to two bottles in their cages: one containing 1% sucrose solution and the other containing water. All mice were acclimated to the two-bottle choice condition for 2 days, with bottle positions changed every 6 h to prevent positional preference. After a 24-h period of food and water deprivation, mice had free access to both bottles for 12 h, after which the positions were swapped. The volumes of water and sucrose solution consumed were recorded, and sucrose preference was calculated as the percentage of sucrose solution consumed relative to total fluid intake.

### Western blotting and lectin blotting

Equal amounts of proteins were separated by sodium dodecyl sulfate-polyacrylamide gel electrophoresis (SDS-PAGE) and transferred to polyvinylidene difluoride membranes (Millipore Sigma) at 10 V for 1 h. For Western blotting, membranes were blocked with 5% nonfat dry milk for 1.5 h at room temperature, incubated with specific primary antibodies overnight at 4° C, and then with appropriate HRP-conjugated secondary antibodies. For lectin blotting, membranes were blocked in 5% BSA for 1.5 h at room temperature, followed by incubation with biotinylated LCA lectin overnight at 4° C. The Vectastain ABC kit (Vector Laboratories) was then used to probe the immunoreactive bands. Specific proteins were visualized using ECL select reagent (Amersham).

### Immunoprecipitation

The MT-360 Micro Tube Mixer was used to combine 1.5 μl anti-gp130 antibody with 15 μl Ab-Capcher MAG2 at 4 °C for 2 hours. After three times of washes with PBS, 500 μg of proteins from each tissue were added and incubated overnight at 4 °C. After two PBS washes, these immunoprecipitates were identified using western blotting and lectin blotting.

### Real-time PCR

RNA was extracted using TRIzol reagent (Invitrogen) and reverse-transcribed (1 μg total RNA) into cDNA using PrimeScript RT reagent with gDNA Eraser (Takara). Primer sequences for real-time PCR are shown in Table 2. Real-time PCR was performed using TB Green Premix Ex Taq II (Tli RNaseH Plus) (Takara) under the following conditions: initial denaturation at 95 °C for 30 s, followed by 40 cycles of denaturation at 95 °C for 5 s and annealing at 60 °C for 30 s.

**Table 2:** Primer sequences for real-time PCR

Target genes	Primer sequences (5′-3′)	
	Forward sequences	Reverse sequences
*Il6*	CTGCAAGAGACTTCCATCCAG	AGTGGTATAGACAGGTCTGTTGG
*TNF-α*	AAGTCAACCTCCTCTCTGCC	CCGGACTCCGCAAAGTCTAA
*Il1b*	TGTCTGAAGCAGCTATGGC	TGTTGATGTGCTGCTGCG
*iNOS*	ATGACTCCCAGCACAAAGGG	AACAGCACTCTCTTGCGGACC
*Iba1*	TCTGAGGAGCTATGAGCC	TCCATGTACTTCGTCTTGAAGGC
*Gapdh*	GTCGTGGAGTCTACTGGTGTCTT	GAGATGATGACCCTTTTGGC
*Fut8*	AACAGAAGCAGCCTTCCACC	TACCGATTGTGTAGTCCAGC

### Immunofluorescence

Mice were deeply anesthetized with sodium pentobarbital and perfused intracardially with 4% paraformaldehyde in 0.01 M phosphate buffer. After perfusion, brains were removed and fixed in 4% paraformaldehyde for 24 h, followed by dehydration in 30% sucrose solution. Brains were frozen and sectioned at 25 μm using a cryostat. Sections were collected in 24-well plates, washed three times with PBS, permeabilized with 0.3% Triton X-100 for 30 min, and blocked with 4% BSA in PBS for 30 min at room temperature. They were then incubated overnight at 4 °C with 1% BSA, 0.3% Triton X-100, and anti-Iba1 antibody (1:500) in PBS. After three washes with PBS, sections were incubated with the goat anti-rabbit antibody Alexa Fluor 488 for 2 h at room temperature. Following three additional washes in PBS, sections were incubated with 4′,6-diamidino-2-phenylindole (DAPI) for 10 min to label cell nuclei. Sections were then transferred to slides, sealed with fluorescence mounting medium, and examined using a ZEISS LSM 900 confocal microscope. The number of Iba1-labeled microglia was quantified using ImageJ software (https://imagej.net/ij/).

### Golgi staining

Golgi staining was performed using FD Rapid GolgiStain Kit (PK401). Following deep anesthesia, the mouse brain was removed and washed with double-distilled water. Brain tissue was treated according to the manufacturer’s protocol. Tissue was sectioned into 100 μm slices, transferred to gelatin-coated microscope slides (catalog no. PO101), and air-dried at room temperature. Sections were rinsed with double-distilled water, stained in the staining solution for 10 min, and then rinsed again. Stained sections were dehydrated in a series of ethanol concentrations (50%, 75%, 95% ethanol, and anhydrous ethanol) and finally cleared with xylene. Mounting with Permount Mounting Medium (E17986–01). Three or four neurons were selected per mice in the CA1 region of the hippocampus, and three mice were used per group. Images were captured using an Olympus IX71 microscope at 10X and 40X magnification. Spine density is defined as the number of protrusions divided by the dendritic shaft length. Total dendritic length and number of branches were measured using ImageJ software.

### Statistical analysis

All data are presented as mean ± SD from at least three independent experiments. Statistical analysis was conducted using a one-way analysis of variance (ANOVA) with Tukey's *post hoc* test *via* GraphPad Prism 5.0 software (GraphPad Software, Inc). A probability value of *p* was considered as follows: n.s. (no significance), *p* > 0.05; ∗*p* < 0.05; ∗∗*p* < 0.01; ∗∗∗*p* < 0.001.

## Data availability

All data presented in the figures and tables, and supplementary information of this paper are available.

## Supporting information

This article contains supporting Information.

## Conflicts of interest

The authors declare that they have no conflicts of interest with the contents of this article.

## References

[1] Zhu Y.J., Huang J., Chen R., Zhang Y., He X., Duan W.X. (2024). Autophagy dysfunction contributes to NLRP1 inflammasome-linked depressive-like behaviors in mice. J. Neuroinflammation.

[2] Kong S., Chen Y., Huang H., Yang W., Lyu D., Wang F. (2024). Efficacy of transcranial direct current stimulation for treating anhedonia in patients with depression: a randomized, double-blind, sham-controlled clinical trial. J. Affect. Disord..

[3] Zu X., Xin J., Xie H., Xu X., Shen Y., Wang J. (2024). Characteristics of gut microbiota and metabolic phenotype in patients with major depressive disorder based on multi-omics analysis. J. Affect. Disord..

[4] Ren F.F., Hillman C.H., Wang W.G., Li R.H., Zhou W.S., Liang W.M. (2024). Effects of aerobic exercise on cognitive function in adults with major depressive disorder: a systematic review and meta-analysis. Int. J. Clin. Health Psychol..

[5] Zhang Y., Lai S., Zhang J., Wang Y., Zhao H., He J. (2024). The effectiveness of vortioxetine on neurobiochemical metabolites and cognitive of major depressive disorders patients: a 8-week follow-up study. J. Affect. Disord..

[6] Yu X., Wang S., Wu W., Chang H., Shan P., Yang L. (2023). Exploring new mechanism of depression from the effects of virus on nerve cells. Cells.

[7] Cui L., Li S., Wang S., Wu X., Liu Y., Yu W. (2024). Major depressive disorder: hypothesis, mechanism, prevention and treatment. Signal Transduct. Target. Ther..

[8] Malhi G.S., Mann J.J. (2018). Depression. Lancet..

[9] Sukoff Rizzo S.J., Neal S.J., Hughes Z.A., Beyna M., Rosenzweig-Lipson S., Moss S.J. (2012). Evidence for sustained elevation of IL-6 in the CNS as a key contributor of depressive-like phenotypes. Transl. Psychiatry.

[10] Wiener C.D., Moreira F.P., Portela L.V., Strogulski N.R., Lara D.R., da Silva R.A. (2019). Interleukin-6 and Interleukin-10 in mood disorders: a population-based study. Psychiatry Res..

[11] Ting E.Y., Yang A.C., Tsai S.J. (2020). Role of interleukin-6 in depressive disorder. Int. J. Mol. Sci..

[12] Ellul P., Boyer L., Groc L., Leboyer M., Fond G. (2016). Interleukin-1 beta-targeted treatment strategies in inflammatory depression: toward personalized care. Acta Psychiatr. Scand..

[13] Lu J., Jin K., Jiao J., Liu R., Mou T., Chen B. (2023). YY1 (Yin-Yang 1), a transcription factor regulating systemic inflammation, is involved in cognitive impairment of depression. Psychiatry Clin. Neurosci..

[14] Yang Y., Gu K., Meng C., Li J., Lu Q., Zhou X. (2023). Relationship between sleep and serum inflammatory factors in patients with major depressive disorder. Psychiatry Res..

[15] Enache D., Pariante C.M., Mondelli V. (2019). Markers of central inflammation in major depressive disorder: a systematic review and meta-analysis of studies examining cerebrospinal fluid, positron emission tomography and post-mortem brain tissue. Brain Behav. Immun..

[16] Dowlati Y., Herrmann N., Swardfager W., Liu H., Sham L., Reim E.K. (2010). A meta-analysis of cytokines in major depression. Biol. Psychiatry.

[17] Kopschina Feltes P., Doorduin J., Klein H.C., Juarez-Orozco L.E., Dierckx R.A., Moriguchi-Jeckel C.M. (2017). Anti-inflammatory treatment for major depressive disorder: implications for patients with an elevated immune profile and non-responders to standard antidepressant therapy. J. Psychopharmacol..

[18] Elizabeth A., Adegbuyi A., Olusegun A., Benneth B.A., Anthony E., Abayomi A. (2020). Morin hydrate attenuates chronic stress-induced memory impairment and degeneration of hippocampal subfields in mice: the role of oxidative, nitrergic and neuroinflammatory pathways. Metab. Brain Dis..

[19] Liu L., Tang J., Liang X., Li Y., Zhu P., Zhou M. (2024). Running exercise alleviates hippocampal neuroinflammation and shifts the balance of microglial M1/M2 polarization through adiponectin/AdipoR1 pathway activation in mice exposed to chronic unpredictable stress. Mol. Psychiatry.

[20] Wang H., Yang Y., Yang S., Ren S., Feng J., Liu Y. (2021). Ginsenoside Rg1 ameliorates neuroinflammation via suppression of Connexin43 ubiquitination to attenuate depression. Front. Pharmacol..

[21] Yirmiya R., Rimmerman N., Reshef R. (2015). Depression as a microglial disease. Trends Neurosci..

[22] Wang H., He Y., Sun Z., Ren S., Liu M., Wang G. (2022). Microglia in depression: an overview of microglia in the pathogenesis and treatment of depression. J. Neuroinflammation.

[23] Li B., Yang W., Ge T., Wang Y., Cui R. (2022). Stress induced microglial activation contributes to depression. Pharmacol. Res..

[24] Li W., Ali T., He K., Liu Z., Shah F.A., Ren Q. (2021). Ibrutinib alleviates LPS-induced neuroinflammation and synaptic defects in a mouse model of depression. Brain Behav. Immun..

[25] Wei H., Yu C., Zhang C., Ren Y., Guo L., Wang T. (2023). Butyrate ameliorates chronic alcoholic central nervous damage by suppressing microglia-mediated neuroinflammation and modulating the microbiome-gut-brain axis. Biomed. Pharmacother..

[26] Kokkosis A.G., Madeira M.M., Hage Z., Valais K., Koliatsis D., Resutov E. (2024). Chronic psychosocial stress triggers microglial-/macrophage-induced inflammatory responses leading to neuronal dysfunction and depressive-related behavior. Glia.

[27] Villar-Portela S., Muinelo-Romay L., Cuevas E., Gil-Martin E., Fernandez-Briera A. (2013). FX enzyme and GDP-L-Fuc transporter expression in colorectal cancer. Histopathology..

[28] Bonin C.P., Freshour G., Hahn M.G., Vanzin G.F., Reiter W.D. (2003). The GMD1 and GMD2 genes of Arabidopsis encode isoforms of GDP-D-mannose 4,6-dehydratase with cell type-specific expression patterns. Plant Physiol..

[29] Yamaguchi Y., Ikeda Y., Takahashi T., Ihara H., Tanaka T., Sasho C. (2000). Genomic structure and promoter analysis of the human alpha1, 6-fucosyltransferase gene (FUT8). Glycobiology.

[30] Ayukawa T., Matsumoto K., Ishikawa H.O., Ishio A., Yamakawa T., Aoyama N. (2012). Rescue of Notch signaling in cells incapable of GDP-L-fucose synthesis by gap junction transfer of GDP-L-fucose in Drosophila. Proc. Natl. Acad. Sci. U. S. A..

[31] Becker D.J., Lowe J.B. (2003). Fucose: biosynthesis and biological function in mammals. Glycobiology.

[32] Adhikari E., Liu Q., Burton C., Mockabee-Macias A., Lester D.K., Lau E. (2022). L-fucose, a sugary regulator of antitumor immunity and immunotherapies. Mol. Carcinog..

[33] Lester D.K., Burton C., Gardner A., Innamarato P., Kodumudi K., Liu Q. (2023). Fucosylation of HLA-DRB1 regulates CD4(+) T cell-mediated anti-melanoma immunity and enhances immunotherapy efficacy. Nat. Cancer.

[34] Feofanova N.A., Bets V.D., Borisova M.A., Litvinova E.A. (2022). L-fucose reduces gut inflammation due to T-regulatory response in Muc2 null mice. PLoS One.

[35] Li W., Wu P., Jin T., Jia J., Chen B., Liu T. (2024). L-fucose and fucoidan alleviate high-salt diet-promoted acute inflammation. Front. Immunol..

[36] Yao H., Shi H., Jiang C., Fan M., Zhang Y., Qian W. (2023). L-Fucose promotes enteric nervous system regeneration in type 1 diabetic mice by inhibiting SMAD2 signaling pathway in enteric neural precursor cells. Cell Commun. Signal..

[37] Hayes A.J., Melrose J. (2018). Glycans and glycosaminoglycans in neurobiology: key regulators of neuronal cell function and fate. Biochem. J..

[38] Alghazwi M., Smid S., Karpiniec S., Zhang W. (2019). Comparative study on neuroprotective activities of fucoidans from Fucus vesiculosus and Undaria pinnatifida. Int. J. Biol. Macromol..

[39] Takahashi M., Kizuka Y., Ohtsubo K., Gu J., Taniguchi N. (2016). Disease-associated glycans on cell surface proteins. Mol. Aspects Med..

[40] Wang X., Gu J., Miyoshi E., Honke K., Taniguchi N. (2006). Phenotype changes of Fut8 knockout mouse: core fucosylation is crucial for the function of growth factor receptor(s). Methods Enzymol..

[41] Hullen A., Falkenstein K., Weigel C., Huidekoper H., Naumann-Bartsch N., Spenger J. (2021). Congenital disorders of glycosylation with defective fucosylation. J. Inherit. Metab. Dis..

[42] Fukuda T., Hashimoto H., Okayasu N., Kameyama A., Onogi H., Nakagawasai O. (2011). Alpha1,6-fucosyltransferase-deficient mice exhibit multiple behavioral abnormalities associated with a schizophrenia-like phenotype: importance of the balance between the dopamine and serotonin systems. J. Biol. Chem..

[43] Gu W., Fukuda T., Isaji T., Hang Q., Lee H.H., Sakai S. (2015). Loss of alpha1,6-fucosyltransferase decreases hippocampal long term potentiation: implications for core fucosylation in the regulation of ampa receptor heteromerization and cellular signaling. J. Biol. Chem..

[44] Guo H., Sun Q., Huang X., Wang X., Zhang F., Qu W. (2024). Fucosyltransferase 8 regulates adult neurogenesis and cognition of mice by modulating the Itga6-PI3K/Akt signaling pathway. Sci. China. Life Sci..

[45] Xu X., Fukuda T., Takai J., Morii S., Sun Y., Liu J. (2024). Exogenous l-fucose attenuates neuroinflammation induced by lipopolysaccharide. J. Biol. Chem..

[46] Lu X., Zhang D., Shoji H., Duan C., Zhang G., Isaji T. (2019). Deficiency of alpha1,6-fucosyltransferase promotes neuroinflammation by increasing the sensitivity of glial cells to inflammatory mediators. Biochim. Biophys. Acta Gen. Subj..

[47] Duman R.S., Aghajanian G.K. (2012). Synaptic dysfunction in depression: potential therapeutic targets. Science.

[48] Allen L., Dwivedi Y. (2020). MicroRNA mediators of early life stress vulnerability to depression and suicidal behavior. Mol. Psychiatry.

[49] Kendler K.S., Karkowski L.M., Prescott C.A. (1999). Causal relationship between stressful life events and the onset of major depression. Am. J. Psychiatry.

[50] Walton N.L., Antonoudiou P., Barros L., Dargan T., DiLeo A., Evans-Strong A. (2023). Impaired endogenous neurosteroid signaling contributes to behavioral deficits associated with chronic stress. Biol. Psychiatry.

[51] Malta M.B., Martins J., Novaes L.S., Dos Santos N.B., Sita L., Camarini R. (2021). Norepinephrine and glucocorticoids modulate chronic unpredictable stress-induced increase in the type 2 CRF and glucocorticoid receptors in brain structures related to the HPA Axis activation. Mol. Neurobiol..

[52] Shin H.S., Lee S.H., Moon H.J., So Y.H., Jang H.J., Lee K.H. (2024). Prolonged stress response induced by chronic stress and corticosterone exposure causes adult neurogenesis inhibition and astrocyte loss in mouse hippocampus. Brain Res. Bull..

[53] Wu H., Denna T.H., Storkersen J.N., Gerriets V.A. (2019). Beyond a neurotransmitter: the role of serotonin in inflammation and immunity. Pharmacol. Res..

[54] Li W., Ali T., Zheng C., Liu Z., He K., Shah F.A. (2021). Fluoxetine regulates eEF2 activity (phosphorylation) via HDAC1 inhibitory mechanism in an LPS-induced mouse model of depression. J. Neuroinflammation.

[55] Wang L., Wang R., Liu L., Qiao D., Baldwin D.S., Hou R. (2019). Effects of SSRIs on peripheral inflammatory markers in patients with major depressive disorder: a systematic review and meta-analysis. Brain Behav. Immun..

[56] Garcia-Garcia M.L., Tovilla-Zarate C.A., Villar-Soto M., Juarez-Rojop I.E., Gonzalez-Castro T.B., Genis-Mendoza A.D. (2022). Fluoxetine modulates the pro-inflammatory process of IL-6, IL-1beta and TNF-alpha levels in individuals with depression: a systematic review and meta-analysis. Psychiatry Res..

[57] Zhang F., Zhou H., Wilson B.C., Shi J.S., Hong J.S., Gao H.M. (2012). Fluoxetine protects neurons against microglial activation-mediated neurotoxicity. Parkinsonism Relat. Disord..

[58] Liu Q.R., Shi C.N., Wang F., Tong J.H. (2023). Neuroinflammation-induced parvalbumin interneuron and oscillation deficits might contribute to neurobehavioral abnormities in a two-hit model of depression. Heliyon.

[59] Liu D., Wang Z., Liu S., Wang F., Zhao S., Hao A. (2011). Anti-inflammatory effects of fluoxetine in lipopolysaccharide(LPS)-stimulated microglial cells. Neuropharmacology.

[60] Wakasugi D., Kondo S., Ferdousi F., Mizuno S., Yada A., Tominaga K. (2024). A rare olive compound oleacein functions as a TrkB agonist and mitigates neuroinflammation both in vitro and in vivo. Cell Commun. Signal..

[61] Albrakati A., Alsharif K.F., Al Omairi N.E., Alsanie W.F., Almalki A.S.A., Abd Elmageed Z.Y. (2021). Neuroprotective efficiency of prodigiosins conjugated with selenium nanoparticles in rats exposed to chronic unpredictable mild stress is mediated through antioxidative, anti-inflammatory, anti-apoptotic, and neuromodulatory activities. Int. J. Nanomedicine..

[62] Wang D., Xu X., Wu Y., Lin Y., Gao M., Hu P. (2019). SMIP004: a compound with antidepressant-like activities in mouse models. Eur. J. Pharmacol..

[63] Wu A., Zhang J. (2023). Neuroinflammation, memory, and depression: new approaches to hippocampal neurogenesis. J. Neuroinflammation.

[64] Tartt A.N., Mariani M.B., Hen R., Mann J.J., Boldrini M. (2022). Dysregulation of adult hippocampal neuroplasticity in major depression: pathogenesis and therapeutic implications. Mol. Psychiatry.

[65] Fuchs E., Czeh B., Kole M.H., Michaelis T., Lucassen P.J. (2004). Alterations of neuroplasticity in depression: the hippocampus and beyond. Eur. Neuropsychopharmacol..

[66] Singhal G., Baune B.T. (2017). Microglia: an interface between the loss of neuroplasticity and depression. Front. Cell. Neurosci..

[67] Xiao K., Luo Y., Liang X., Tang J., Wang J., Xiao Q. (2021). Beneficial effects of running exercise on hippocampal microglia and neuroinflammation in chronic unpredictable stress-induced depression model rats. Transl. Psychiatry.

[68] Schramm E., Waisman A. (2022). Microglia as central protagonists in the chronic stress response. Neurol. Neuroimmunol. Neuroinflamm..

[69] Imai Y., Kohsaka S. (2002). Intracellular signaling in M-CSF-induced microglia activation: role of Iba1. Glia.

[70] Yu L., Huang L., Zhao Y., Liu S., Zhou R., Yue Y. (2024). Atorvastatin promotes pro/anti-inflammatory phenotypic transformation of microglia via wnt/beta-catenin pathway in hypoxic-ischemic neonatal rats. Mol. Neurobiol..

[71] Bennett M.L., Bennett F.C., Liddelow S.A., Ajami B., Zamanian J.L., Fernhoff N.B. (2016). New tools for studying microglia in the mouse and human CNS. Proc. Natl. Acad. Sci. U. S. A..

[72] Liang Y., Aditi, Onyoni F., Wang H., Gonzales C., Sunyakumthorn P. (2023). Brain transcriptomics reveal the activation of neuroinflammation pathways during acute Orientia tsutsugamushi infection in mice. Front. Immunol..

[73] Hamsalakshmi, Alex A.M., Arehally Marappa M., Joghee S., Chidambaram S.B. (2022). Therapeutic benefits of flavonoids against neuroinflammation: a systematic review. Inflammopharmacology.

[74] Almutabagani L.F., Almanqour R.A., Alsabhan J.F., Alhossan A.M., Alamin M.A., Alrajeh H.M. (2023). Inflammation and treatment-resistant depression from clinical to animal study: a possible link?. Neurol. Int..

[75] Hu Y., Zhang X., Zhang J., Xia X., Li H., Qiu C. (2021). Activated STAT3 signaling pathway by ligature-induced periodontitis could contribute to neuroinflammation and cognitive impairment in rats. J. Neuroinflammation.

[76] Millot P., San C., Bennana E., Porte B., Vignal N., Hugon J. (2020). STAT3 inhibition protects against neuroinflammation and BACE1 upregulation induced by systemic inflammation. Immunol. Lett..

[77] Zheng Z.V., Chen J., Lyu H., Lam S.Y.E., Lu G., Chan W.Y. (2022). Novel role of STAT3 in microglia-dependent neuroinflammation after experimental subarachnoid haemorrhage. Stroke Vasc. Neurol..

[78] Zhou K., Chen J., Wu J., Wu Q., Jia C., Xu Y.X.Z. (2019). Atractylenolide III ameliorates cerebral ischemic injury and neuroinflammation associated with inhibiting JAK2/STAT3/Drp1-dependent mitochondrial fission in microglia. Phytomedicine.

[79] Tian Y.S., Zhong D., Liu Q.Q., Zhao X.L., Sun H.X., Jin J. (2018). Upregulation of miR-216a exerts neuroprotective effects against ischemic injury through negatively regulating JAK2/STAT3-involved apoptosis and inflammatory pathways. J. Neurosurg..

[80] Mojiri-Forushani H., Khajehali E., Adelipour M., Mohammadi A. (2023). Inhibitory effects of fluoxetine on the secretion of inflammatory mediators and JAK/STAT3 and JNK/TLR4 gene expression. Mol. Biol. Rep..

[81] Zanders L., Kny M., Hahn A., Schmidt S., Wundersitz S., Todiras M. (2022). Sepsis induces interleukin 6, gp130/JAK2/STAT3, and muscle wasting. J. Cachexia Sarcopenia Muscle.

[82] Hussain S.F., Kong L.Y., Jordan J., Conrad C., Madden T., Fokt I. (2007). A novel small molecule inhibitor of signal transducers and activators of transcription 3 reverses immune tolerance in malignant glioma patients. Cancer Res..

[83] Hatiboglu M.A., Kong L.Y., Wei J., Wang Y., McEnery K.A., Fuller G.N. (2012). The tumor microenvironment expression of p-STAT3 influences the efficacy of cyclophosphamide with WP1066 in murine melanoma models. Int. J. Cancer.

[84] Zaccard C.R., Gippo I., Song A., Geula C., Penzes P. (2023). Dendritic spinule-mediated structural synaptic plasticity: implications for development, aging, and psychiatric disease. Front. Mol. Neurosci..

[85] Bai Z., Gao T., Zhang R., Lu Y., Tian J., Wang T. (2023). Inhibition of IL-6 methylation by Saikosaponin C regulates neuroinflammation to alleviate depression. Int. Immunopharmacol..

[86] Popova D., Castren E., Taira T. (2017). Chronic fluoxetine administration enhances synaptic plasticity and increases functional dynamics in hippocampal CA3-CA1 synapses. Neuropharmacology.

[87] Djordjevic A., Djordjevic J., Elakovic I., Adzic M., Matic G., Radojcic M.B. (2012). Fluoxetine affects hippocampal plasticity, apoptosis and depressive-like behavior of chronically isolated rats. Prog. Neuropsychopharmacol. Biol. Psychiatry.

[88] Bijata M., Baczynska E., Muller F.E., Bijata K., Masternak J., Krzystyniak A. (2022). Activation of the 5-HT7 receptor and MMP-9 signaling module in the hippocampal CA1 region is necessary for the development of depressive-like behavior. Cell Rep..

[89] Egashira Y., Suganuma M., Kataoka Y., Higa Y., Ide N., Morishita K. (2019). Establishment and characterization of a fucosylated alpha-fetoprotein-specific monoclonal antibody: a potential application for clinical research. Sci. Rep..

[90] Feichtinger R.G., Hullen A., Koller A., Kotzot D., Grote V., Rapp E. (2021). A spoonful of L-fucose-an efficient therapy for GFUS-CDG, a new glycosylation disorder. EMBO Mol. Med..

[91] Kudo T., Fujii T., Ikegami S., Inokuchi K., Takayama Y., Ikehara Y. (2007). Mice lacking alpha1,3-fucosyltransferase IX demonstrate disappearance of Lewis x structure in brain and increased anxiety-like behaviors. Glycobiology.

[92] Guo L.T., Wang S.Q., Su J., Xu L.X., Ji Z.Y., Zhang R.Y. (2019). Baicalin ameliorates neuroinflammation-induced depressive-like behavior through inhibition of toll-like receptor 4 expression via the PI3K/AKT/FoxO1 pathway. J. Neuroinflammation.

[93] Dong S.Q., Zhang Q.P., Zhu J.X., Chen M., Li C.F., Liu Q. (2018). Gypenosides reverses depressive behavior via inhibiting hippocampal neuroinflammation. Biomed. Pharmacother..

[94] Zhao Q., Wu X., Yan S., Xie X., Fan Y., Zhang J. (2016). The antidepressant-like effects of pioglitazone in a chronic mild stress mouse model are associated with PPARgamma-mediated alteration of microglial activation phenotypes. J. Neuroinflammation.

[95] Tynan R.J., Naicker S., Hinwood M., Nalivaiko E., Buller K.M., Pow D.V. (2010). Chronic stress alters the density and morphology of microglia in a subset of stress-responsive brain regions. Brain Behav. Immun..

[96] Bian Y., Pan Z., Hou Z., Huang C., Li W., Zhao B. (2012). Learning, memory, and glial cell changes following recovery from chronic unpredictable stress. Brain Res. Bull..

[97] Stockmeier C.A., Mahajan G.J., Konick L.C., Overholser J.C., Jurjus G.J., Meltzer H.Y. (2004). Cellular changes in the postmortem hippocampus in major depression. Biol. Psychiatry.

[98] Du Preez A., Onorato D., Eiben I., Musaelyan K., Egeland M., Zunszain P.A. (2021). Chronic stress followed by social isolation promotes depressive-like behaviour, alters microglial and astrocyte biology and reduces hippocampal neurogenesis in male mice. Brain Behav. Immun..

[99] Hsu M.P., Frausto R., Rose-John S., Campbell I.L. (2015). Analysis of IL-6/gp130 family receptor expression reveals that in contrast to astroglia, microglia lack the oncostatin M receptor and functional responses to oncostatin M. Glia.

[100] Khandaker G.M., Pearson R.M., Zammit S., Lewis G., Jones P.B. (2014). Association of serum interleukin 6 and C-reactive protein in childhood with depression and psychosis in young adult life: a population-based longitudinal study. JAMA Psychiatry.

[101] Hodes G.E., Pfau M.L., Leboeuf M., Golden S.A., Christoffel D.J., Bregman D. (2014). Individual differences in the peripheral immune system promote resilience versus susceptibility to social stress. Proc. Natl. Acad. Sci. U. S. A..

[102] Rebelo A.L., Gubinelli F., Roost P., Jan C., Brouillet E., Van Camp N. (2021). Complete spatial characterisation of N-glycosylation upon striatal neuroinflammation in the rodent brain. J. Neuroinflammation.

[103] Mueller T.M., Yates S.D., Haroutunian V., Meador-Woodruff J.H. (2017). Altered fucosyltransferase expression in the superior temporal gyrus of elderly patients with schizophrenia. Schizophr. Res..

[104] Cvetko A., Kifer D., Gornik O., Klaric L., Visser E., Lauc G. (2020). Glycosylation alterations in multiple sclerosis show increased proinflammatory potential. Biomedicines.

[105] Chen C.C., Engelborghs S., Dewaele S., Le Bastard N., Martin J.J., Vanhooren V. (2010). Altered serum glycomics in Alzheimer disease: a potential blood biomarker?. Rejuvenation Res..

[106] Boeck C., Pfister S., Burkle A., Vanhooren V., Libert C., Salinas-Manrique J. (2018). Alterations of the serum N-glycan profile in female patients with major depressive disorder. J. Affect. Disord..

[107] Wang X., Inoue S., Gu J., Miyoshi E., Noda K., Li W. (2005). Dysregulation of TGF-beta1 receptor activation leads to abnormal lung development and emphysema-like phenotype in core fucose-deficient mice. Proc. Natl. Acad. Sci. U. S. A..

[108] Gao C., Maeno T., Ota F., Ueno M., Korekane H., Takamatsu S. (2012). Sensitivity of heterozygous alpha1,6-fucosyltransferase knock-out mice to cigarette smoke-induced emphysema: implication of aberrant transforming growth factor-beta signaling and matrix metalloproteinase gene expression. J. Biol. Chem..

[109] Sosicka P., Ng B.G., Pepi L.E., Shajahan A., Wong M., Scott D.A. (2022). Origin of cytoplasmic GDP-fucose determines its contribution to glycosylation reactions. J. Cell Biol..

[110] Sun Y., Xu X., Wu T., Fukuda T., Isaji T., Morii S. (2024). Core fucosylation within the Fc-FcgammaR degradation pathway promotes enhanced IgG levels via exogenous L-fucose. J. Biol. Chem..

[111] Erritzoe D., Godlewska B.R., Rizzo G., Searle G.E., Agnorelli C., Lewis Y. (2023). Brain serotonin release is reduced in patients with depression: a [(11)C]Cimbi-36 positron emission tomography study with a d-amphetamine challenge. Biol. Psychiatry.

[112] Sullivan G.M., Oquendo M.A., Milak M., Miller J.M., Burke A., Ogden R.T. (2015). Positron emission tomography quantification of serotonin(1A) receptor binding in suicide attempters with major depressive disorder. JAMA Psychiatry.

[113] You Y.H., Qin Z.Q., Zhang H.L., Yuan Z.H., Yu X. (2019). MicroRNA-153 promotes brain-derived neurotrophic factor and hippocampal neuron proliferation to alleviate autism symptoms through inhibition of JAK-STAT pathway by LEPR. Biosci. Rep..

[114] Henrik Heiland D., Ravi V.M., Behringer S.P., Frenking J.H., Wurm J., Joseph K. (2019). Tumor-associated reactive astrocytes aid the evolution of immunosuppressive environment in glioblastoma. Nat. Commun..

[115] Wang L., Zhao D., Wang H., Wang L., Liu X., Zhang H. (2021). FPS-ZM1 inhibits LPS-induced microglial inflammation by suppressing JAK/STAT signaling pathway. Int. Immunopharmacol..

[116] Ji Y., Li M., Chang M., Liu R., Qiu J., Wang K. (2022). Inflammation: roles in skeletal muscle atrophy. Antioxidants (Basel).

[117] Milaneschi Y., Simmons W.K., van Rossum E.F.C., Penninx B.W. (2019). Depression and obesity: evidence of shared biological mechanisms. Mol. Psychiatry.

[118] Kong E., Sucic S., Monje F.J., Savalli G., Diao W., Khan D. (2015). STAT3 controls IL6-dependent regulation of serotonin transporter function and depression-like behavior. Sci. Rep..

[119] Kwon S.H., Han J.K., Choi M., Kwon Y.J., Kim S.J., Yi E.H. (2017). Dysfunction of microglial STAT3 alleviates depressive behavior via neuron-microglia interactions. Neuropsychopharmacology.

[120] Holmes S.E., Abdallah C., Esterlis I. (2023). Imaging synaptic density in depression. Neuropsychopharmacology.

[121] Shang C., Yao R.M., Guo Y., Ding Z.C., Sun L.J., Ran Y.H. (2020). Translocator protein-mediated fast-onset antidepressant-like and memory-enhancing effects in chronically stressed mice. J. Psychopharmacol..

[122] Li N., Liu R.J., Dwyer J.M., Banasr M., Lee B., Son H. (2011). Glutamate N-methyl-D-aspartate receptor antagonists rapidly reverse behavioral and synaptic deficits caused by chronic stress exposure. Biol. Psychiatry.

[123] Nicolas C.S., Peineau S., Amici M., Csaba Z., Fafouri A., Javalet C. (2012). The Jak/STAT pathway is involved in synaptic plasticity. Neuron.

[124] Xiao S., Zhang Y., Liu Z., Li A., Tong W., Xiong X. (2023). Alpinetin inhibits neuroinflammation and neuronal apoptosis via targeting the JAK2/STAT3 signaling pathway in spinal cord injury. CNS Neurosci. Ther..

[125] Balschun D., Wetzel W., Del Rey A., Pitossi F., Schneider H., Zuschratter W. (2004). Interleukin-6: a cytokine to forget. FASEB J..

[126] Sparrow N.A., Anwar F., Covarrubias A.E., Rajput P.S., Rashid M.H., Nisson P.L. (2021). IL-6 inhibition reduces neuronal injury in a murine model of ventilator-induced lung injury. Am. J. Respir. Cell Mol. Biol..

[127] Matthies H., Staak S., Krug M. (1996). Fucose and fucosyllactose enhance in-vitro hippocampal long-term potentiation. Brain Res..

[128] Hayashi M., Takai J., Yu L., Motohashi H., Moriguchi T., Yamamoto M. (2015). Whole-body in vivo monitoring of inflammatory diseases exploiting human interleukin 6-luciferase transgenic mice. Mol. Cell. Biol..

