# Supplementary material for: Exogenous L-fucose attenuates depression induced by chronic unpredictable stress: Implicating core fucosylation has an antidepressant potential
Source: J Biol Chem. 2025 Jan 27;301(3):108230. doi: 10.1016/j.jbc.2025.108230 (PMC11879694; doi:10.1016/j.jbc.2025.108230)
Supplement: Supplemental Figures [file mmc1.pdf]

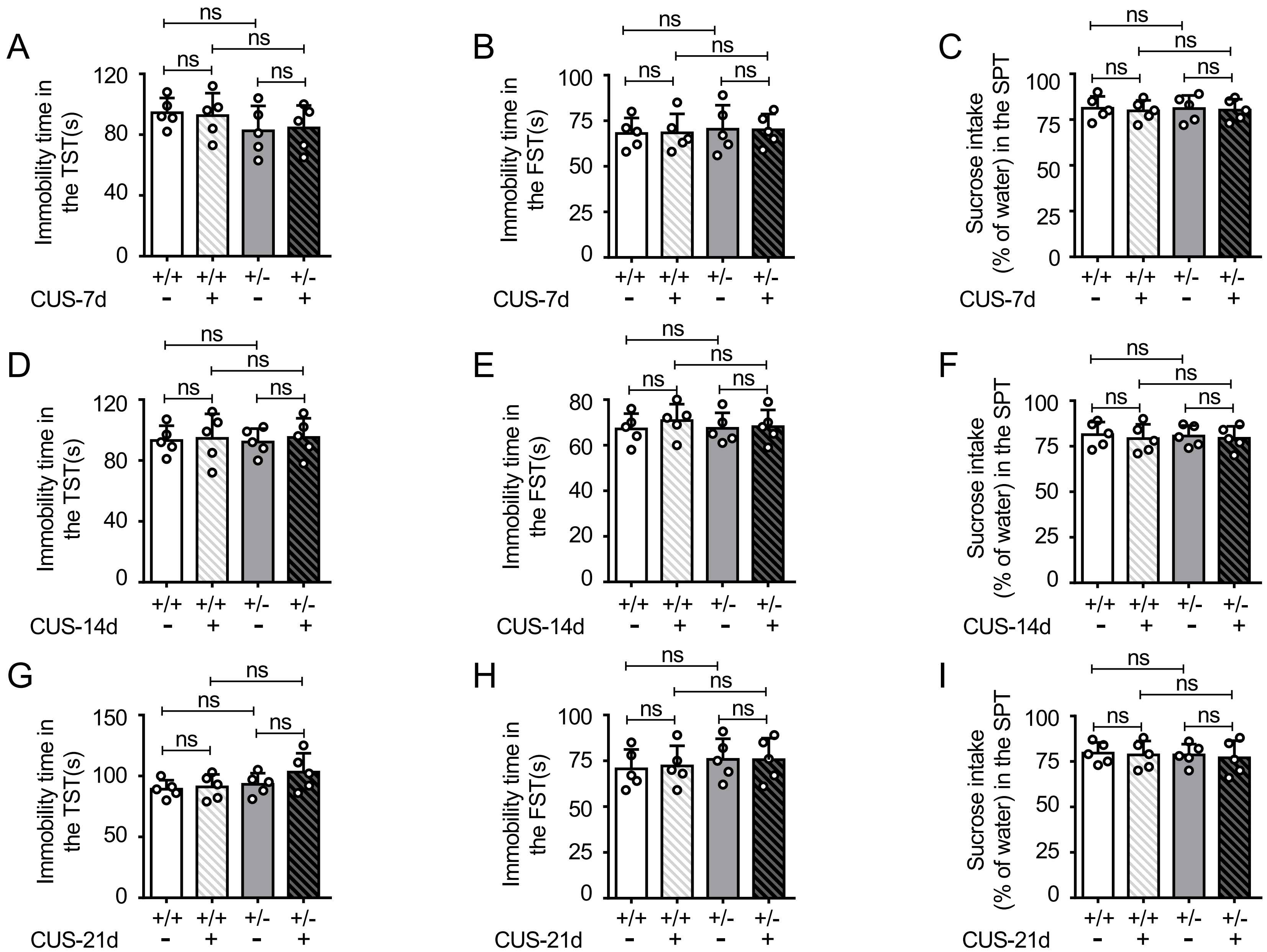

sFig.1. Depressive-like behaviors of *Fut8*$^{+/+}$ and *Fut8*$^{+/-}$ mice at 7, 14, and 21 days of CUS.

The immobility time of mice in the tail suspension test (TST) after 7, 14, and 21 days of CUS were shown in panels A, D, and G, respectively. The immobility time of mice in the forced swimming test (FST) after 7, 14, and 21 days of CUS were shown in panels B, E, and H, respectively. The sucrose consumption of mice in the sucrose preference test (SPT) after 7, 14, and 21 days of CUS were shown in panels C, F, and I, respectively. Data were analyzed by one-way ANOVA with Tukey's multiple comparison tests and showed as the mean $\pm$ SD. n = 5 mice per group. n.s. $p > 0.05$.

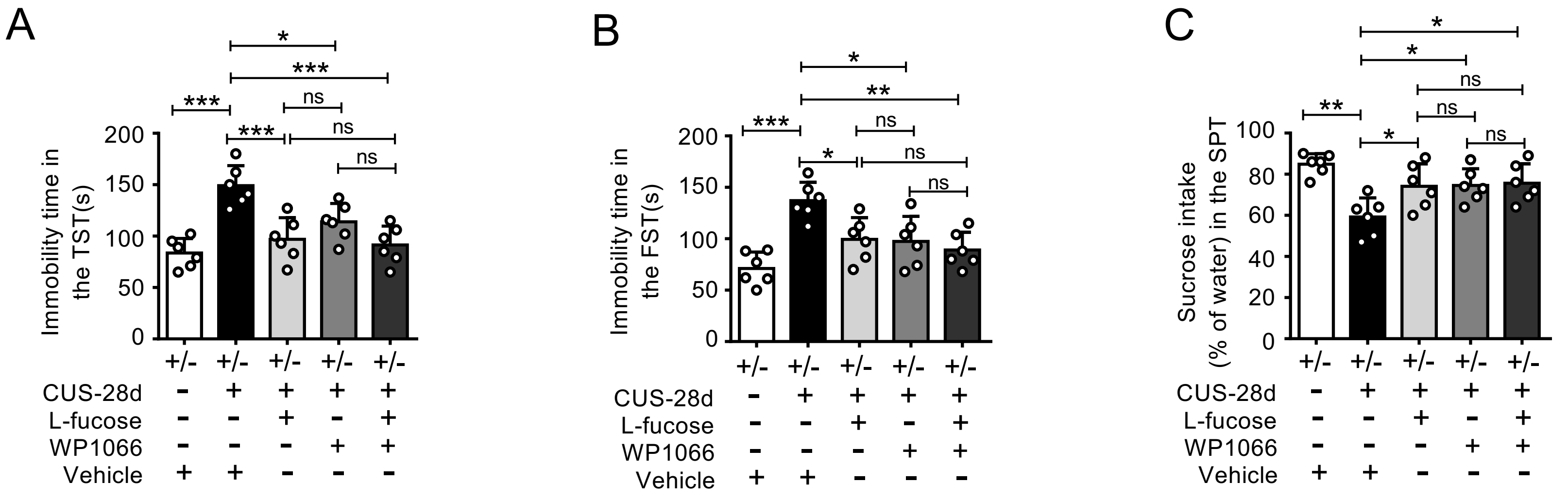

sFig.2. Effects of WP1066, JAK2/STAT3 inhibitor, on depression like behaviors of *Fut8*$^{+/-}$ mice exposed to CUS. Following a 28-day exposure to the CUS model, *Fut8*$^{+/-}$ mice were given L-fucose, WP1066, or the combination of L-fucose and WP1066 and then tested for depressive behavior as described in the Experimental procedures. (A) The immobility time of mice in the tail suspension test (TST) after 28 days of CUS. (B) The immobility time of mice in the forced swimming test (FST) after 28 days of CUS. (C) The sucrose consumption of mice in the sucrose preference test (SPT) after 28 days of CUS. Data were analyzed by one-way ANOVA with Tukey's multiple comparison tests and showed as the mean ± SD. n = 6 mice per each group. n.s. $p > 0.05$; *$p < 0.05$; **$p < 0.01$; ***$p < 0.001$.

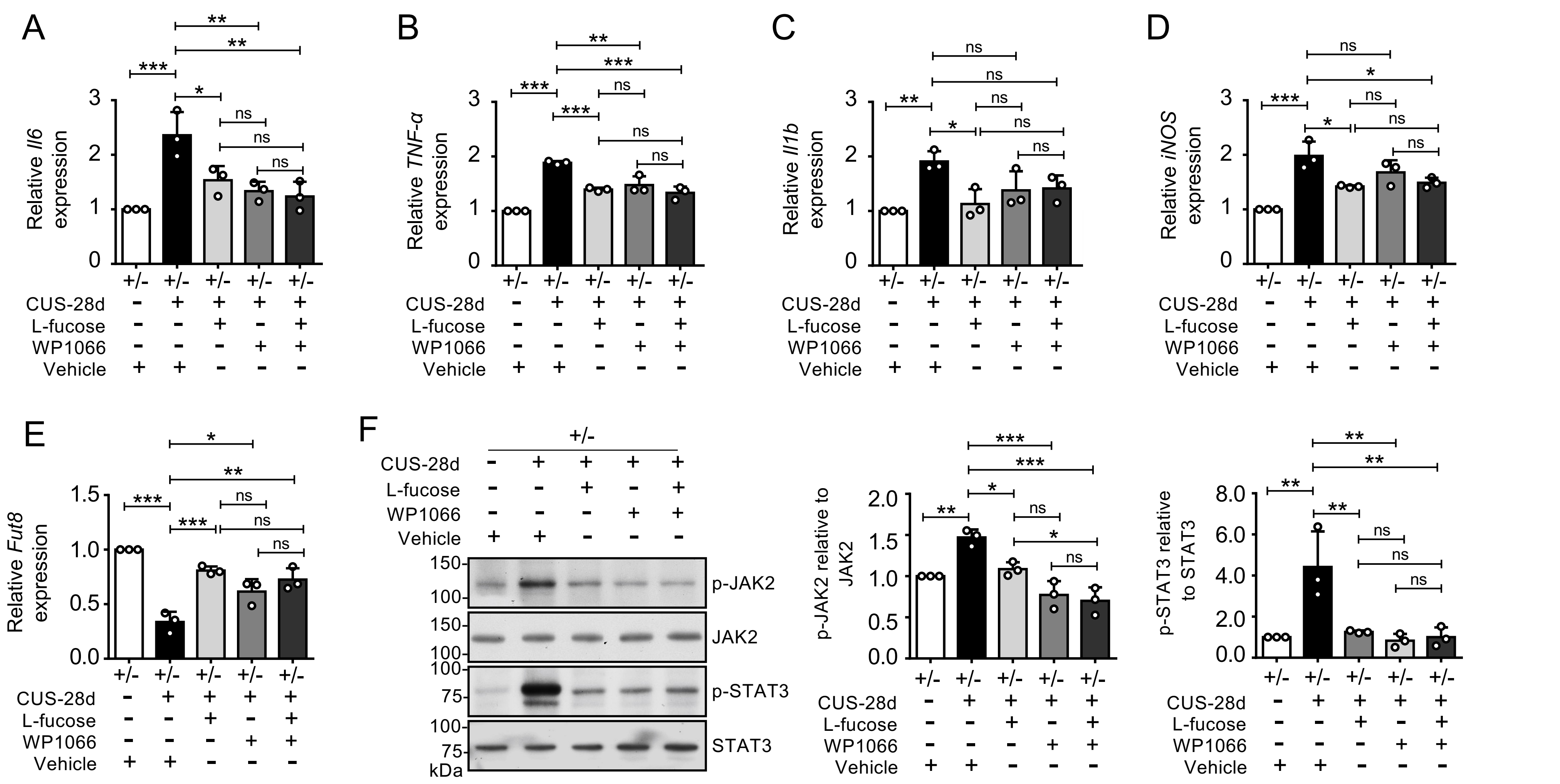

sFig.3. Effects of WP1066 on inflammatory cytokines, *Fut8* mRNA levels and JAK2/STAT3 signaling pathway.

Fut8$^{+/-}$ mice were exposed to the CUS model for 28 days and treated with L-fucose, WP1066, or the combination of L-fucose and WP1066. (A-E) The mRNA levels of *Il6*, *TNF-α, Il1b, iNOS,* or *Fut8* in the hippocampal tissues were detected by real-time PCR, as shown in panels A, B, C, D, and E, respectively. *Gapdh* was used as an internal control. Values were normalized to that of the *Gapdh*. The values of *Il6*, *TNF-α*, *Il1b*, *iNOS*, or *Fut8 versus Gapdh* in vehicle-injected mice were set to 1.0. The one-way ANOVA test with Tukey's multiple comparison test was used to calculate the quantitative data, displayed as the mean ± SD of three independent experiments. n = 3 mice per group. n.s. $p > 0.05$; *$p < 0.05$; **$p < 0.01$; ***$p < 0.001$, (F) The phosphorylation levels of JAK2 and STAT3 in the hippocampal tissues were detected by western blotting. The ratios of p-JAK2 *versus* JAK2 or p-STAT3 *versus* STAT3 in vehicle-injected mice were set to 1.0. The quantitative data were calculated using the one-way ANOVA with Tukey's multiple comparison test and presented as the mean ± SD of three independent experiments. n = 3 mice per group. n.s. $p > 0.05$; *$p < 0.05$; **$p < 0.01$; ***$p < 0.001$.

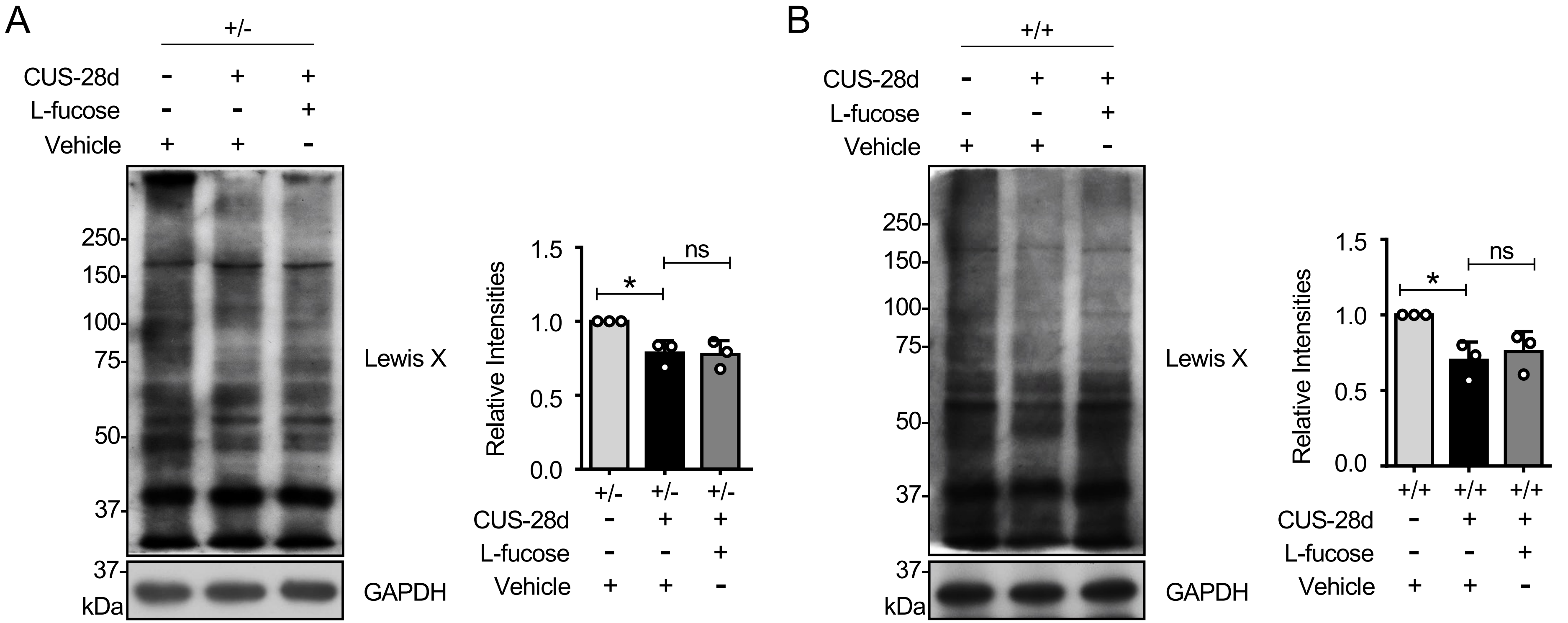

sFig.4. Effects of exogenous L-fucose administration on Lewis X epitope expression in $\boldsymbol{Fut8^{+/-}}$ **and** $\boldsymbol{Fut8^{+/+}}$ **mice exposed to CUS.** The Lewis X epitope's expression levels in mice's hippocampus tissues were examined by western blotting using anti-Lewis X antibody in $Fut8^{+/-}$ (A) and $Fut8^{+/+}$ mice (B). The loading control was GAPDH. The ratio of Lewis X to GAPDH in $Fut8^{+/-}$ or $Fut8^{+/+}$ mice given vehicle was set to 1.0. Data for all bands were analyzed by one-way ANOVA with Tukey's multiple comparison test and are presented as mean ± SD of three independent experiments. n = 3 mice per group. n.s. $p > 0.05$; $*p < 0.05$.

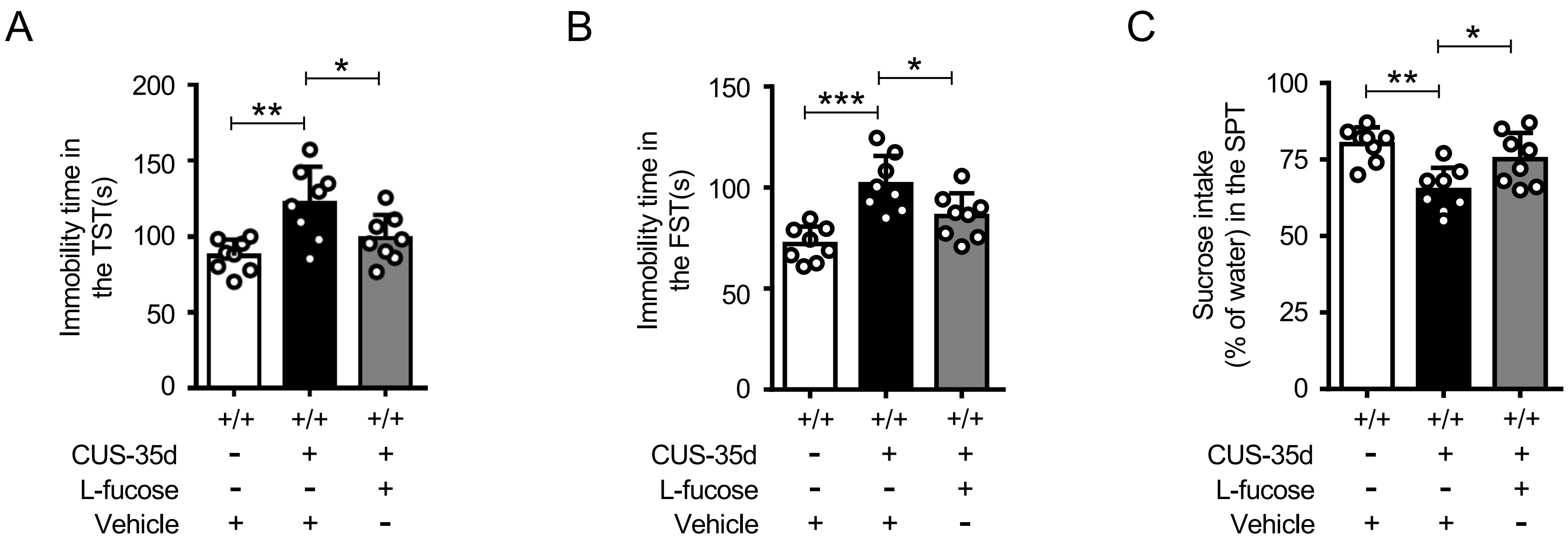

sFig.5. L-fucose reversed the depressive-like behavior of *Fut8*$^{+/+}$ mice after 35 days of CUS.

Fut8$^{+/+}$ mice were subjected to the CUS model for 35 days and treated with L-fucose. (A) The immobility time of mice in tail suspension test (TST) after 35 days of CUS. (B) The immobility time of mice in forced swimming test (FST) after 35 days of CUS. (C) The sucrose consumption of mice in the sucrose preference test (SPT) after 35 days of CUS. Data was analyzed by one-way ANOVA with Tukey's multiple comparison tests and showed as the mean ± SD. n = 8 mice per group. *$p < 0.05$; **$p < 0.01$; ***$p < 0.001$.
